# Divide and conquer: genetics, mechanism, and evolution of the ferrous iron transporter Feo in *Helicobacter pylori*

**DOI:** 10.3389/fmicb.2023.1219359

**Published:** 2023-07-04

**Authors:** Camilo Gómez-Garzón, Shelley M. Payne

**Affiliations:** ^1^Department of Molecular Biosciences, University of Texas at Austin, Austin, TX, United States; ^2^John Ring LaMontagne Center for Infectious Disease, The University of Texas at Austin, Austin, TX, United States

**Keywords:** *Vibrio cholerae*, Fur, NikR, nickel, operon, *Helicobacter pylori*, Feo, iron transport

## Abstract

**Introduction:**

Feo is the most widespread and conserved system for ferrous iron uptake in bacteria, and it is important for virulence in several gastrointestinal pathogens. However, its mechanism remains poorly understood. Hitherto, most studies regarding the Feo system were focused on Gammaproteobacterial models, which possess three *feo genes* (*feoA*, *B*, and *C*) clustered in an operon. We found that the human pathogen *Helicobacter pylori* possesses a unique arrangement of the feo genes, in which only *feoA* and *feoB* are present and encoded in distant loci. In this study, we examined the functional significance of this arrangement.

**Methods:**

Requirement and regulation of the individual *H. pylori* feo genes were assessed through *in vivo* assays and gene expression profiling. The evolutionary history of feo was inferred via phylogenetic reconstruction, and AlphaFold was used for predicting the FeoA-FeoB interaction.

**Results and Discussion:**

Both *feoA* and *feoB* are required for Feo function, and feoB is likely subjected to tight regulation in response to iron and nickel by Fur and NikR, respectively. Also, we established that *feoA* is encoded in an operon that emerged in the common ancestor of most, but not all, helicobacters, and this resulted in *feoA* transcription being controlled by two independent promoters. The *H. pylori* Feo system offers a new model to understand ferrous iron transport in bacterial pathogens.

## 1. Introduction

Iron acquisition is a major challenge for bacterial pathogens inside the host, and it is often a determining factor for infection and disease. Most of the iron in the host environment is tightly bound to proteins, hence not readily available for pathogens. Bacterial pathogens have evolved a diverse arsenal of systems for iron acquisition. This includes the secretion of siderophores, organic molecules that bind iron with high affinity. In response to infection and inflammation, the mammalian host may sequester iron and reduce its availability to the pathogen, a pathway known as nutritional immunity (Skaar, 2010; Barber and Elde, 2015).

Iron can exist in two forms, the oxidized state of iron, ferric iron (Fe^3+^), or the reduced state, ferrous iron (Fe^2+^). The more insoluble ferric form is commonly found in association with proteins and siderophores, while ferrous iron can exist as a free ion, especially under conditions of acidic pH and low oxygen tension, such as those present in the gastric tract (Mey et al., 2021). For this reason, the acquisition of free Fe^2+^ may be particularly relevant for bacterial gastric pathogens, and Feo constitutes the most widespread and conserved Fe^2+^ transporter in bacteria (Lau et al., 2016; Sestok et al., 2018).

**Figure 1:**
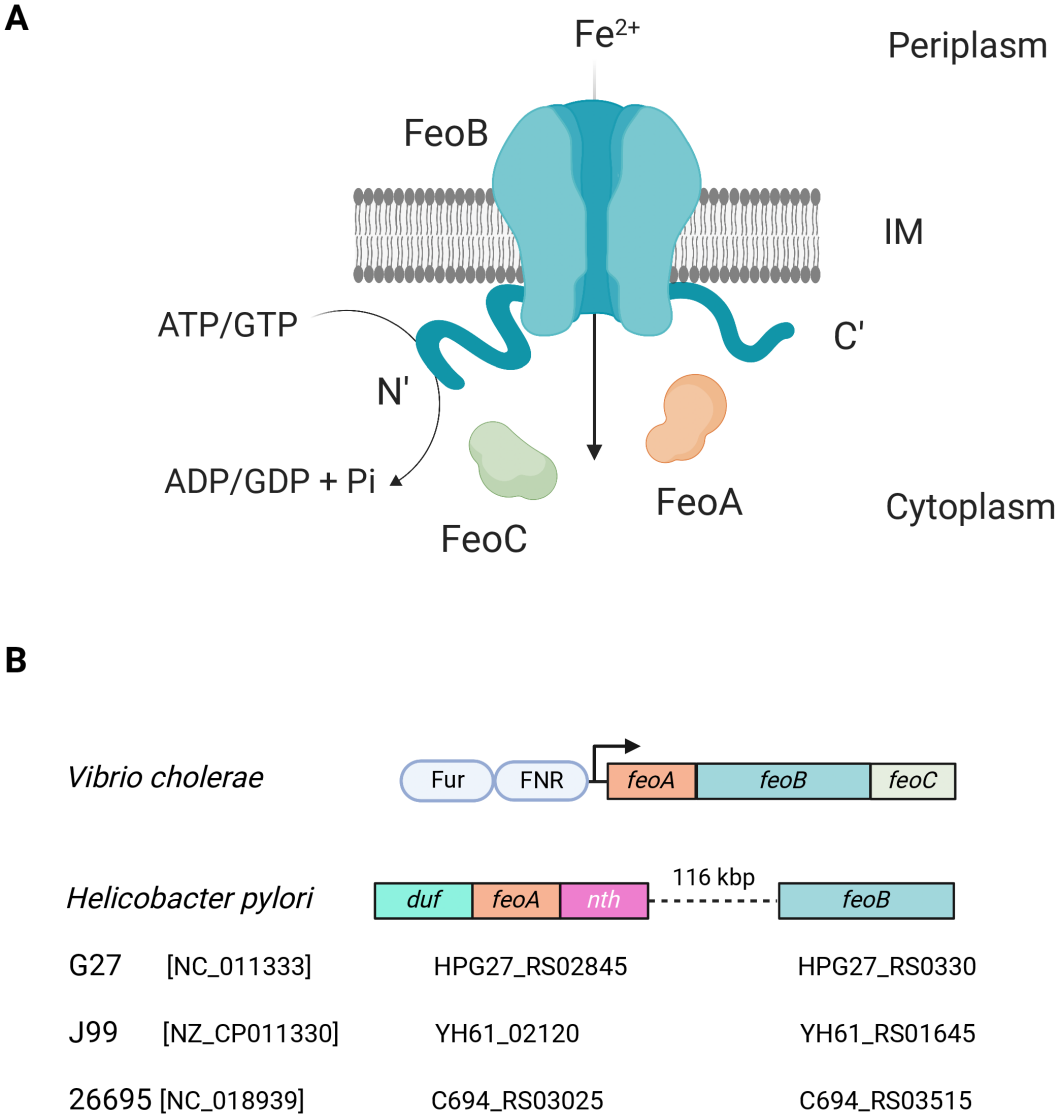
Current understanding of the Feo system. **(A)** Feo works as a large (>250 kDa) complex embedded in the inner membrane (IM), likely consisting of trimers of FeoABC units. The N- and C-terminal domains of FeoB remain in the cytoplasm, and the N-terminal domain has ATPase and GTPase activity, which is essential for Fe^2+^ uptake. This model is largely based on observations made in *V. cholerae* (Gómez-Garzón and Payne, 2020). **(B)** In *V. cholerae*—as well as in most of the Gammaproteobacteria group—the *feo* genes form an operon controlled by Fur and FNR (shown as pale blue ovals). Strikingly, in *H. pylori* the operon architecture is not conserved. Instead, *feoA* is located between *duf* and *nth*, and *feoB* is separated from *feoA* by about 116 kbp. Diagrams are not to scale. The locus tags of *feoA* and *feoB* for three representative *H. pylori* strains are shown below the diagram. The RefSeq accession number of each genome is shown in brackets. Figure created with BioRender.com.

Feo has been shown to contribute to virulence in numerous plant (Franza and Expert, 2013), animal, and human pathogens (Lau et al., 2016), including *Xanthomonas oryzae* (Pandey and Sonti, 2010), *Salmonella enterica* (Boyer et al., 2002), *Legionella pneumophila* (Robey and Cianciotto, 2002), and *Helicobacter pylori* (Velayudhan et al., 2000). Although this system is ubiquitous in bacteria, and important for virulence in some instances, its mechanism and the specific role of its components remain poorly understood. Most of the studies of the Feo system are based on Gammaproteobacteria [Pseudomonadota.] In these species, Feo is made up of three components: FeoA, FeoB, and FeoC, that have been shown to work as a polyprotein complex embedded in the inner membrane in the *V. cholerae* model (Figure 1A). FeoB is a large (~ 85 kDa) transmembrane protein with cytoplasmic N- and C-terminal domains; and its N-terminal domain (NFeoB) is an NTPase that shares homology with eukaryotic G-proteins, such as the human oncogene protein p21 Ras (Lau et al., 2016; Sestok et al., 2018). The catalytic activity of NFeoB is essential for the function of the transporter, and its nucleotide specificity varies among species; for example, NFeoB is a dual GTP/ATPase in *V. cholerae* and *H. pylori*, but solely a GTPase in *E. coli* (Shin et al., 2019, 2020). The other two components, FeoA and FeoC, are small (~8.5 kDa) cytoplasmic proteins with unknown functions though they are both required for iron uptake via Feo (Weaver et al., 2013).

In Gammaproteobacteria, the *feoA, B,* and *C* genes are encoded in an operon controlled by the Ferric Uptake Regulator (Fur) and the Fumarate and Nitrate Reduction regulatory protein (FNR), bacterial master regulators responsive to Fe^2+^ and O_2_, respectively. In previous studies of *V. cholerae*, we have determined that the Feo proteins likely work in a 1:1:1 stoichiometric ratio (Gómez-Garzón and Payne, 2020). We have also shown that FeoA is essential for the assembly of the complex, while FeoC, though not required for complex formation, is critical for function, as those complexes assembled in the absence of FeoC do not support iron uptake (Weaver et al., 2013; Stevenson et al., 2016).

Although research on the bacterial Feo system has primarily focused on Gammaproteobacteria species, alternative architectures of Feo have been identified in other groups (Sestok et al., 2018). For example, the commensal species *Bacteroides fragilis* has a single Feo protein containing a fusion of FeoA and FeoB homolog domains (Sestok et al., 2022). A common feature found when comparing the Feo system among species is that FeoA and FeoB orthologs (or their corresponding domains) are nearly universal, while FeoC is poorly conserved, being present in about 5% of bacterial proteomes, predominantly within the Gamma group (Gómez-Garzón et al., 2022). The specific role of FeoC as well as the functional significance of the different architectures of the Feo system are still to be determined.

By exploring these diverse architectures of Feo among bacteria, we found that *H. pylori*—an important human pathogen for its causal relationship with peptic ulcers and gastric cancer (Herrera and Parsonnet, 2009; Plummer et al., 2015; de Martel et al., 2020)—exhibits a unique arrangement of the *feo* genes. *Feo* is not an operon in this species, since *feoA* and *feoB* are separated by 116 kbp. *feoB* has canonical Fur-binding boxes in its putative promoter, while *feoA* is embedded between two genes in an operon-like arrangement with no evident Fur binding boxes (Figure 1B). Namely, *feoA* localizes between the *nth* gene (downstream), which encodes the endonuclease III, and an upstream gene annotated as a “hypothetical Domain of Unknown Function (DUF) 3,971-containing protein.” *H. pylori* is naturally competent and recombination events drive evolution of subpopulations within the host during infection (Blaser and Berg, 2001; Suerbaum and Josenhans, 2007; Linz et al., 2014; Kobayashi, 2016; Jackson et al., 2020). Consequently, *H. pylori* is characterized by having a highly plastic genome, with low synteny and the absence of several transcription factors commonly found in other species. For instance, *H. pylori* lacks FNR, and it is not rare that it lacks the operon structure of several systems, which is the case for *feo*. Thus, *feoA* was initially overlooked because it was not associated with *feoB*. In consequence, although FeoB likely contributes to virulence in *H. pylori*, whether *feoA* is also required for Fe^2+^ uptake remains unclear.

Characterizing the Feo system in *H. pylori* offers a new model in addition to that of Gammaproteobacteria to understand this major bacterial iron transporter. Equally important, this represents a model of Feo relevant for an important human pathogen. In this study, we conducted an initial characterization of the *H. pylori* Feo system. We determined the requirement of *feoA*; complex formation by FeoA and B; and transcriptional regulation of both genes, including the role of the transcriptional regulator Fur. Additionally, we modeled the evolutionary history of this *feo* architecture in the context of other helicobacters and the Campylobacterota [Epsilonproteobacteria] group.

## 2. Materials and methods

### 2.1. Reagents, bacterial strains, and growth conditions

All reagents and growth media were purchased from Sigma-Aldrich Chemical Company unless stated otherwise. *E. coli* and *V. cholerae* strains were routinely grown in Luria-Bertani (LB) broth (10 g/L tryptone, 5 g/L yeast extract, and 10 g/L NaCl in double-distilled water) or on LB agar (1.5% w/v bacteriological agar) at 37°C and 200 rpm for liquid media. These strains were preserved at −80°C in tryptic soy broth (TSB) with 20% glycerol.

*Helicobacter pylori* strains were grown and maintained following the protocols described in Whitmire and Merrell (2012): Freezer stocks were prepared in brain heart infusion (BHI) broth supplemented with 20% v/v glycerol and 10% v/v fetal bovine serum (FBS). For culturing in solid media, *H. pylori* was always grown in horse blood agar (HBA) plates containing 4% w/v Columbia agar base (BD Difco™), 5% v/v defibrinated horse blood (Remel^™^), 0.2% w/v β-cyclodextrin, and antibiotic supplementation (5 μg/mL trimethoprim, 8 μg/mL amphotericin B, 10 μg/mL vancomycin hydrochloride, 5 μg/mL cefsulodin sodium salt, and 0.33 μg/mL Polymyxin B Sulfate) at 37°C under microaerobic atmosphere. For liquid cultures, brucella broth (BB) supplemented with 10% v/v FBS and 10 μg/mL vancomycin was used. Cells were grown at 37°C with 100 rpm shaking under microaerobic atmosphere. In both cases, microaerobiosis was generated with CampyGen™ packets in a Oxoid^™^ AnaeroJar^™^ system (Thermo Fisher Scientific).

For strains harboring plasmids, antibiotics were used as follows: For *E. coli*, 50 μg/mL ampicillin, 8 μg/mL chloramphenicol, and 12.5 μg/mL tetracycline; for *V. cholerae*, 25 μg/mL ampicillin and 6.25 μg/mL tetracycline; and for *H. pylori*, 25 μg/mL kanamycin and 8 μg/mL chloramphenicol.

Bacterial strains and plasmids used in this study are listed in Supplementary Tables S1, S2, respectively.

### 2.2. Cloning of *Helicobacter pylori feo* genes

Primers used in this study are listed in Supplementary Table S3. The accession numbers for the *H. pylori* loci used for primer design are listed in Supplementary Table S4 PCRs were all done using high-fidelity Phusion *Taq* polymerase (New England BioLabs). Restriction and ligation reactions were carried out using NEB restriction enzymes and NEB T4 ligase, respectively. Plasmids were routinely purified using a QIAprep Spin Miniprep Kit by Qiagen. All constructs were initially cloned into *E. coli* TOP10 via CaCl_2_ heat-shock transformation. To confirm the sequence and directionality of the DNA constructs, the final products were submitted to Genewiz for Sanger DNA sequencing, and results were analyzed with the SnapGene v6.1.2 software.

For experiments in *E. coli* and *V. cholerae* purified genomic DNA from *H. pylori* 26695 (ATCC^®^) was used as a template to amplify *HpfeoA* with the primers HpFeoA-EcoRI-F and HpFeoA-EcoRI-R, and *HpfeoB* with the primers HpFeoB-EcoRI-F and HpFeoB-NotI-R. To generate pHpfeoA, the PCR product for *HpfeoA* was digested with *Eco*RI and cloned into the corresponding restriction site in pACYC184 in the same direction as the P*cam*^r^ promoter. Similarly, the PCR product for *HpfeoB* was digested with *Eco*RI and *Bam*HI and cloned into the corresponding restriction sites in pWKS30 in the same direction as the P*lac* promoter to generate pHpfeoB.

The complementation vector for *HpfeoA* (pTMHpfeoA) in *H. pylori* G27 was constructed by substituting the *gfp* gene in pTM117 with *HpfeoA* from this strain. Briefly, *gfp* in pTM117 was removed by digestion with *BamH*I and *Pst*I to then insert the *HpfeoA* coding sequence, which was previously amplified from *H. pylori* G27 genomic DNA—purified from overnight liquid cultures using a PureLink^™^ Genomic DNA Kit (Thermo Fisher Scientific)—with the primers HpFeoA-BamHI-F and HpFeoA-PstI-R, and treated with the same restriction enzymes.

### 2.3. Growth assessment of *Vibrio cholerae* EPV6

*Vibrio cholerae* EPV6 was transformed with equimolar amounts of pACYC184 and pWKS30 (or derivatives) simultaneously by electroporation as previously described (Occhino et al., 1998). EPV6 cells carrying the Feo constructs under analysis were streaked onto different quadrants of LB agar plates, with and without 10 μM heme supplementation, and incubated at 37°C overnight. In these assays, functional Feo systems and empty vectors were used as positive and negative controls, respectively. To facilitate development of isolated colonies after 24 h, plates without heme were supplemented with 20 μM FeSO_4_ stabilized with 5 mM sodium ascorbate. For replication, all these assays were carried out in at least three separate plates under each condition; each plate was inoculated with individual colonies. Observable growth after incubation was considered a positive result.

### 2.4. DNA manipulations in *Helicobacter pylori*

*feoA* and *feoB H. pylori* isogenic deletion mutants were generated following the protocol described in Servetas et al. (2021). Specifically, each *feo* gene was disrupted via homologous recombination by transforming *H. pylori* G27 with splicing by overlap extension (SOE) PCR products containing the *kan^r^* marker with homologous flanking sequences for the *feo* gene. SOE PCR products for *HpfeoA* and *HpfeoB* were kindly donated by Dr. Nina Salama (Fred Hutch Cancer Center, Seattle, WA).

In short, 30–50 μL of concentrated *H. pylori* cell suspension prepared from 24 h liquid cultures are spotted onto prewarmed HBA plates and dried for 3–4 h at 37°C under microaerobiosis. Afterwards, 50–100 ng of the SOE PCR product are added on top of the spotted cells and incubated for 24 h at 37°C in microaerobic conditions. Then, cells are swabbed and resuspended in liquid media, and this suspension is plated onto HBA plates containing kanamycin. Plates are incubated at 37°C under microaerobiosis and continuously monitored for colony growth. Colonies that grew after 3–5 days were isolated and streaked on kanamycin-containing HBA plates. Successful recombination was confirmed by amplifying the *feo* loci with the primers Conf-HpfeoA-F and Conf-HpfeoA-R (for *HpfeoA*::*kan^r^*) and Conf-HpfeoB-F and Conf-HpfeoB-R (for *HpfeoB*::*kan^r^*) and sequenced to verify the insertion of the *kan^r^* marker.

For insertion of plasmids in *H. pylori* G27, the same transformation protocol described above was followed using a plasmid prep as the DNA source. Since all plasmid transformations in *H. pylori* were made with pTM117 derivatives, selection was done in HBA plates supplemented with kanamycin.

### 2.5. Assessment of nickel sensitivity in *Helicobacter pylori*

Nickel sensitivity of *H. pylori feo* mutants was assessed by growing them on HBA plates containing a gradient of Ni^2+^ concentration up to 250 μM. In order to expose the cells to the whole gradient, 50 μL of a concentrated cell suspension (prepared from a 24 h liquid culture) was spotted on the lowest-concentrated border of each plate and the plate was tilted to let the drop slip. Gradient plates were prepared by pouring 0 and 250 μM melted HBA separately on Petri dishes, as described in Weinberg (1959). Plain HBA plates (no Ni^2+^) and *H. pylori* WT were used as controls in this experiment. Likewise, a complementation strain for *HpfeoA* was also included to rule out polar effects of the *HpfeoA* deletion.

### 2.6. Construction of promoter fusions and GFP reporter assay

For expression in *E. coli*, transcriptional reporters (pGT- vectors as listed in Supplementary Table S2) were constructed by cloning the putative promoters under study upstream of the promoterless *gfp* gene in pGTXN3, between the *Bam*HI and *Xma*I sites (for P*feoA* and P*feoB*) or *Xma*I and *Xba*I (for P*duf*). Promoters were obtained by PCR from *H. pylori* G27 genomic DNA with the primers promHpFeoA-XmaI-F and promHpFeoA-BamHI-R for P*feoA*, promHpFeoB-XmaI-F and promHpFeoB-BamHI-R for P*feoB*, and prom-DUF-XmaI-F and prom-DUF-XbaI-R for P*duf*. Equivalent transcriptional reporters compatible with *H. pylori* (pTM- vectors in Table X) were constructed in pTM117 with the same cloning strategy.

For *gfp* expression assays the protocol described in Carpenter et al. (2007) was followed with some modifications: *H. pylori* G27 and Δ*fur* were grown for 48 h in liquid culture as described above Then 1.5 mL of each culture were washed twice with phosphate-buffered saline (PBS) and used in an SDS-PAGE gel to be immunoblotted with anti-GFP antibodies (JL-8 from Clonetech).

### 2.7. C-FLAG epitope tagging

A C-terminal *Pst*I restriction site was added in the *HpfeoA* CDS in pHp*feoA* via QuikChange site directed mutagenesis (Xia et al., 2015) with the primers HpFeoA-PstC-F and HpFeoA-PstC-R (Supplementary Table S3).

Separately, a dsDNA probe carrying the epitope FLAG coding sequence with *Pst*I sticky ends was generated by annealing the C-FLAG-PstI-Top and C-FLAG-PstI-Btm fragments. The annealed FLAG-coding probe was ligated with the *Pst*I-treated pHp*feoA* plasmid and transformed into *E. coli* TOP10. The final product of this ligation is referred to as pHpfeoA^C-FLAG^.

### 2.8. SDS-PAGE and immunoblotting

Protein samples from cell cultures were analyzed through 8–16% gradient SDS polyacrylamide gels prepared as described in Miller et al. (2016).

For immunoblot analysis, resolved proteins were tank-transferred from the polyacrylamide gel to an Immobilon^®^-P PVDF membrane (Merck Millipore). Then, GFP or FLAG-tagged proteins were detected using mouse monoclonal anti-GFP (JL-8 from Clonetech) or anti-FLAG monoclonal antibody (M2 from Sigma Aldrich), respectively; and visualized using horseradish peroxidase (HRP)-conjugated goat anti-mouse IgG (Bio-Rad) followed by detection with Pierce^™^ ECL Western Blotting Substrate (Thermo Fisher Scientific). To ensure even levels of loading, total protein content in the immunoblotted samples was assessed by Coomassie staining using R-250 Brilliant Blue (Bio-Rad).

### 2.9. RNA isolation, reverse transcription, and quantitative PCR

Iron depletion was induced with a rapid exposure to 2,2′-dipyridyl (dpp) adapted from the method described in Carpenter et al. (2007): *H. pylori* G27 and Δ*fur* were grown overnight, cultures were divided in halves, and one half of each culture was exposed to 200 μM dpp for 1 h. After this incubation, RNA was isolated as described below.

Total RNA was isolated from ∼10^9^ cells using RNA-Bee (Tel-Test, Inc.) per the manufacturer’s instructions. RNA was treated with TURBO™ DNase (Thermo Fisher Scientific) and then precipitated with cold ethanol and resuspended in diethyl pyrocarbonate (DEPC)-treated water (Thermo Fisher Scientific). Integrity of the isolated RNA was checked by electrophoresis in an agarose gel and the concentration, assessed with a NanoDrop® machine (Thermo Fisher Scientific). The RNA was retrotranscribed to cDNA using a SuperScript III system with random hexamers (Thermo Fisher Scientific). Additional reactions using water instead of retrotranscriptase were done and included in both PCR and qPCR runs as a negative control. cDNA was used as a template for PCR and qPCR amplifications.

For regular PCR amplification, a 1:2 cDNA dilution was used as a template. The primers used for obtaining the products shown below were: for A, RTq-junct-F and RTq-junct-R; for B, Duf-feoA-Locus-F and Duf-feoA-Locus-R; and for C, feoA-nth-F and feoA-nth-R. Amplifications were additionally performed on genomic DNA samples as a positive control.

qPCR was conducted with cDNA diluted in a 1:10 ratio with Power SYBR green (Thermo Fisher Scientific) in an Applied Biosystems ViiA 7 instrument with the following parameters: 50°C for 2 min and 95°C for 10 min; followed by 95°C for 15 s and 60°C for 1 min for 40 cycles, with the fluorescence recorded at 60°C. A melting curve was generated as follows: 90°C for 15 s, 60°C for 1 min, and then 95°C for 15 s with the fluorescence recorded every 0.05 s. Relative expression levels were calculated using the threshold cycle (ΔΔ*C_T_*) method (Schmittgen and Livak, 2008). Each reaction produced only one melting curve, indicating that only one target had been amplified during the qPCR reaction. A single amplification product from these reactions was further verified by resoling the samples after the qPCR run through regular agarose electrophoresis.

All of these analyses used *rpoD* and *gyrB* as internal references. The primers used for qPCR were designed using the Primer3 algorithm (Untergasser et al., 2012) optimizing the parameters as recommended by the SYBR green manufacturer: RTq-HpfeoA-F and RTq-HpfeoA-R for *HpfeoA*, RTq-HpfeoB-F and RTq-HpfeoB-R for *HpfeoB*, for RTq-junct-F and RTq-junct-R the *HpfeoA*-*B* junction (junct), RTq-duf-F and RTq-duf-R for *duf*, RTq-pfr-F and RTq-pfr-R for *pfr*, RTq-rpoD-F and RTq-rpoD-R for *rpoD*, and RTq-gyrB-F and RTq-gyrB-R for *gyrB*.

### 2.10. *In vivo* crosslinking

For *in vivo* crosslinking of EPV6 cells, the protocol described in Stevenson et al. (2016) was followed with minor modifications: 50 mL of LB broth supplemented with the appropriate antibiotics were inoculated with overnight cultures in a 1:100 ratio. The culture was grown until mid-log phase (i.e., OD_650_ ≈ 0.6) at 37°C and 200 rpm. Cells were pelleted and washed twice with 25 mL of PBS. All centrifugations were done at 8,500 × *g* for 5 min. Cells were then treated with 25 mL of 0.6% v/v formaldehyde in PBS at room temperature for 6 min with gentle shaking. Then, the reaction was quenched by washing the cells with 10 mL 1.25 M glycine in PBS. Cells were washed with 25 mL PBS to remove the quenching solution, and the final cell pellet was frozen at −80°C until further use.

### 2.11. Cell fractionation

Cell pellets collected and preserved as described above were thawed on ice and resuspended in 5 mL of low-salt buffer, consisting of 100 mM sodium phosphate (pH 7.2), 10% v/v glycerol, 1 mM EDTA, 1 mM PMSF. Samples were sonicated to induce cell lysis, and cell debris was removed by centrifugation at 12,000 × *g* for 10 min. Total membrane pellets were separated from the cytoplasmic fractions at 50,000 rpm for 45 min using a TLA-100.3 rotor (Beckman Coulter). Total membrane pellets were washed twice with high-salt buffer (20 mM sodium phosphate (pH 7.2), 2 M KCl, 10% v/v glycerol, 1 mM EDTA, 1 mM PMSF) and once with 20 mM HEPES-NaOH buffer (pH 7.5) containing 1 mM PMSF. Both cytoplasmic and membrane fractions were preserved at −80°C for further processing.

### 2.12. Mass spectrometry analysis

Cytoplasmic and membrane fractions were first enriched in FLAG-tagged proteins via immunoprecipitation with the same anti-FLAG monoclonal antibody used for immunoblot analyses and SureBeads^TM^ Protein G Magnetic Beads (Bio-Rad) following the manufacturer’s instructions.

Immunoprecipitated samples were subsequently resolved via SDS-PAGE and stained with R-250 Brilliant Blue for no longer than 15 min. The desired bands were excised from the gel, and stored at 4°C in 500 μL of a 10% v/v methanol, 7.5% v/v acetic acid solution until processing.

Protein identification was provided by the UT Austin Center for Biomedical Research Support Biological Mass Spectrometry Facility (RRID: SCR_021728). Proteins were reduced with DTT and alkylated with iodoacetamide, then digested in-gel with trypsin and desalted with Millipore μ-C18 ZipTip pipette tips. Peptide samples were run by LC–MS/MS on a Thermo Ultimate 3000 RSLCnano UPLC in-line with an Orbitrap Fusion Tribrid mass spectrometer. The analytical column was a 75 μm × 25 cm Acclaim PepMap100 C18 column (Thermo Fisher Scientific). The data were collected with FT MS followed by data-dependent acquisition of ion trap MS/MS. Raw data were searched using Proteome Discoverer 2.5 via Sequest HT search engine using 10 ppm mass tolerance for the MS from the FT detector and 0.6 Da for MS/MS from the ion trap detector with fixed modification of carbamidomethylation of cysteine, and variable modifications of methionine oxidation, protein N-terminal acetylation, and protein N-terminal acetylation with Met loss. Validation with Proteome Software Scaffold 5 used a protein threshold of 99% confidence for 2 peptides at a peptide threshold of 1% FDR.

### 2.13. Motif binding analysis

A 200 bp upstream of *feoA*, *feoB*, or *duf* CDS in representative genomes were used to construct the alignment for each *feo* architecture, i.e., operon or separate genes. These alignments were used as inputs for motif discovery and identification with the XSTREME algorithm (Grant and Bailey, 2021) through the MEME Suite (Bailey et al., 2015). CollecTF (Kiliç et al., 2016) was used as a database for annotating discovered motifs together with binding sequences for Fur and NikR reported in the literature (Delany et al., 2005; Arnold et al., 2012; Pich et al., 2012; Carpenter et al., 2013; Agriesti et al., 2014; Roncarati et al., 2016), which were introduced manually. All the other parameters were used with the values set by default.

### 2.14. Phylogenetic inferences

Amino acid sequences for RpoB and RpoC were concatenated when necessary and aligned using MUSCLE through MEGA X (Kumar et al., 2018) with all settings defined by default. The resulting alignments were used to construct the corresponding phylogenetic trees by the maximum likelihood method with the Jones-Taylor-Thornton model and the gamma distribution for evolutionary rates. Cutoff values were fixed at 95% for coverage, and trees were tested by bootstrapping with 300 replicates. Trees were plotted using iTOL v6 (Letunic and Bork, 2021).

The genomic context of each *feoA* gene was determined by manual inspection of the corresponding representative genome listed in Supplementary Table S4.

### 2.15. 3D protein structure modeling

The HpFeoA-HpFeoB interaction was modeled using AlphaFold-Multimer (Evans et al., 2022) through the ChimeraX interface (Pettersen et al., 2021).

## 3. Results

### 3.1. *feoA* is functional and necessary for ferrous iron transport in *Helicobacter pylori*

**Figure 2:**
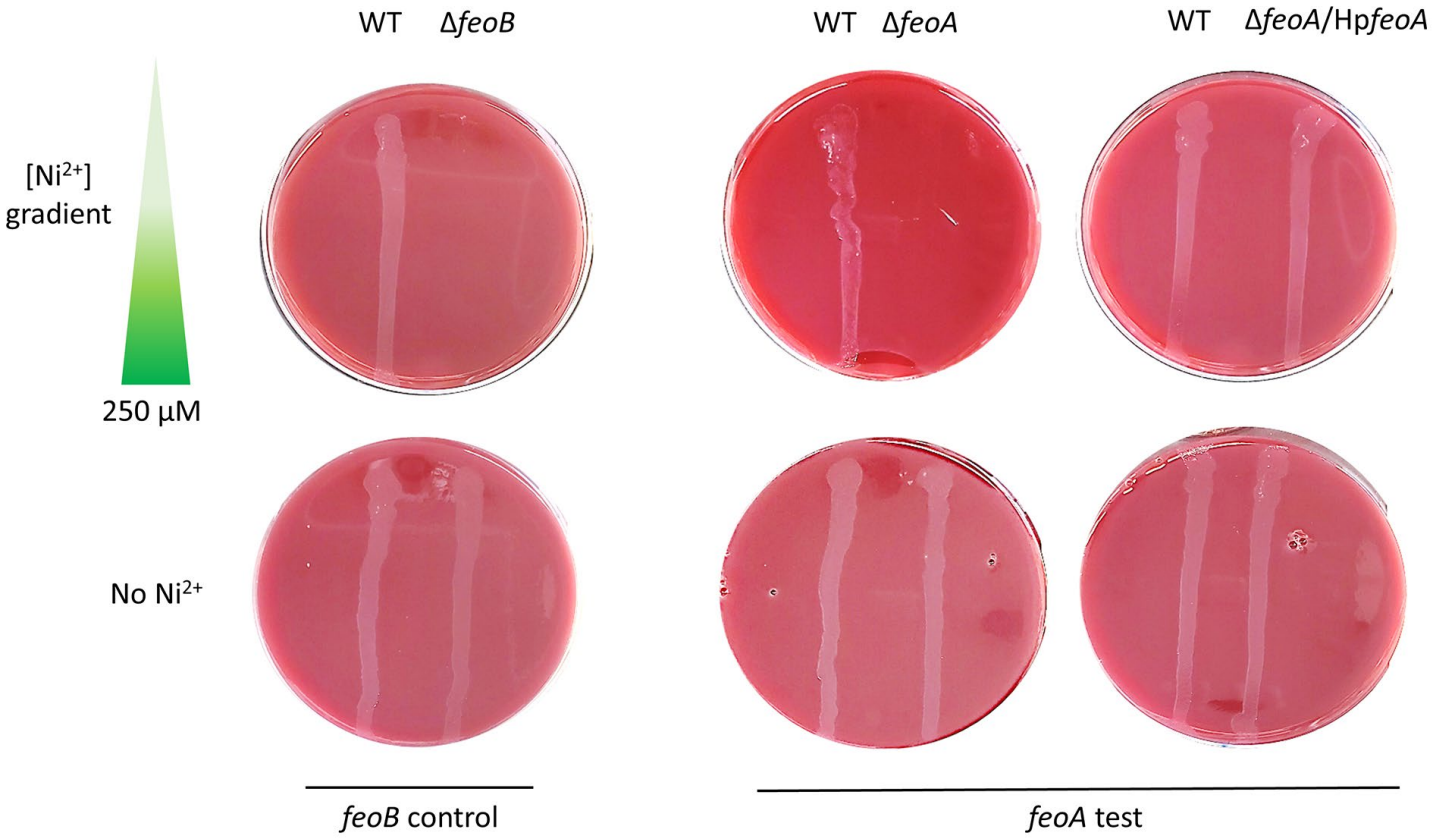
*HpfeoA* and *HpfeoB* are necessary and sufficient to support iron uptake. *V. cholerae* EPV6 requires heme supplementation to grow on LB agar in the absence of a functional iron transport system. EPV6 cells co-transformed with plasmids carrying *HpfeoA* and/or *HpfeoB* (pHpfeoA and pHpfeoB) were streaked on medium with (left panel) or without (right panel) heme. Expression of *V. cholerae feo* genes from the same backbones (pVcfeoA and pVcfeoBC) and the empty vectors in EPV6 served as positive and negative controls, respectively. The data are representative of multiple independent experiments with different transformants (biological replicates).

We examined the architecture and distribution of the *feo* genes in *H. pylori*. The separation of *feoA* from *feoB* as well as the association of *feoA* with the *nth* and *duf* genes are features conserved among *H. pylori* strains (Figure 1B). FeoB is required for ferrous iron transport in *H. pylori* (Velayudhan et al., 2000) and in some species, FeoB is sufficient for function and does not require accessory proteins like FeoA or FeoC (Lau et al., 2016; Sestok et al., 2018; Gómez-Garzón et al., 2022). To determine whether *feoA* is required for Feo function in *H. pylori*, we tested whether both genes are necessary to support iron uptake in the *feo*-null mutant strain *V. cholerae* EPV6. This strain harbors mutations in multiple iron transport systems, and it is therefore unable to grow in standard media such as LB agar, unless the medium is supplied with heme, for which it retains a functional transporter (Peng et al., 2016). Transforming EPV6 with a plasmid carrying a functional Feo system restores growth in non-supplemented media. We have extensively used EPV6 as a tool to unambiguously assess Feo function from diverse species. We cloned the *H. pylori feoA* and *feoB* genes (*HpfeoA* and *HpfeoB*) in separate, compatible vectors. We found that EPV6 is able to grow in non-supplemented medium only when both *HpfeoA* and *HpfeoB* are expressed; the presence of either *HpfeoA* or *HpfeoB* in the absence of the other gene did not support EPV6 growth (Figure 2). We obtained a similar result using *E. coli* H1771, an alternative indicator strain for iron transport (Hantke, 1987). In this strain, both *HpfeoA* and *HpfeoB* were required to alleviate iron starvation (Supplementary Figure S1). These results suggest that *HpfeoA* and *HpfeoB* are necessary and sufficient to assemble a functional iron transporter.

**Figure 3:**
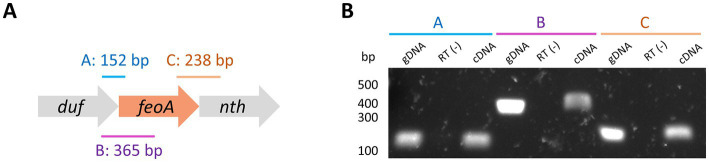
Deletion of *feoA* in *H. pylori* leads to increased sensitivity toward nickel. Similar to Δ*feoB* (left panel), a Δ*feoA* mutant (right panel) failed to grow on Ni^2+^ gradient HBA plates. Complementation of *HpfeoA* from a vector (pTMHpfeoA) restored the WT phenotype in the Δ*feoA* mutant. All strains were able to grow on HBA plates without Ni^2+^ as shown in the bottom section of both panels. These plates correspond to a single representative experiment of multiple biological replicates.

In order to directly assess the function of *H. pylori feo* genes in *H. pylori* G27, we constructed derivative Δ*feoA* and Δ*feoB* mutants, and determined their phenotypes. Deletion of *feoB* in *H. pylori* results in increased sensitivity to heavy metals, especially nickel (Ni^2+^) (Velayudhan et al., 2000). If both *HpfeoA* and *HpfeoB* participate in the same iron transport pathway, deletion of *feoA* should also lead to increased sensitivity to Ni^2+^. We tested this by comparing Ni^2+^ tolerance between Δ*feoA* and Δ*feoB* mutants of *H. pylori* G27. Consistent with this hypothesis, both deletion mutants exhibited increased sensitivity toward Ni^2+^ compared to the wild-type (WT) strain. Specifically, these mutants were unable to grow throughout the concentration gradient tested (up to 250 μM), while the WT strain did not show any inhibition in this range. In the absence of Ni^2+^, both deletion mutants grew similarly to the WT. *In trans* expression of *feoA* from a plasmid restored growth in media containing Ni^2+^ (Figure 3), demonstrating that the observed phenotype of Δ*feoA* was not due to polar effects induced by the gene deletion.

### 3.2. *HpfeoA* belongs to an uncharacterized operon

Because *feoA* is an essential part of the *H. pylori* Feo system but is not linked to *feoB* as it is in most species, we wanted to determine how its genomic context affected expression. In *H. pylori*, *feoA* localizes between the endonuclease III-coding gene (*nth*) and an uncharacterized protein-coding gene (*duf*) as depicted in Figure 1B. The three genes in this cluster are oriented in the same direction and have no intergenic space between them, suggesting the three genes are in an operon. To explore this possibility, we first examined the transcription units in the *H. pylori* G27 genome as annotated in two publicly available repositories, BioCyc (ID number TU2BRX-234) (Karp et al., 2019) and ProOpDB (operon containing the locus tag HPSH112_04095, Supplementary Dataset S1; Taboada et al., 2012). These databases implement different, and independently developed, pipelines for operon inference, and they both predict the *nth*-*feoA*-*duf* cluster to be a single transcription unit.

**Figure 4:**
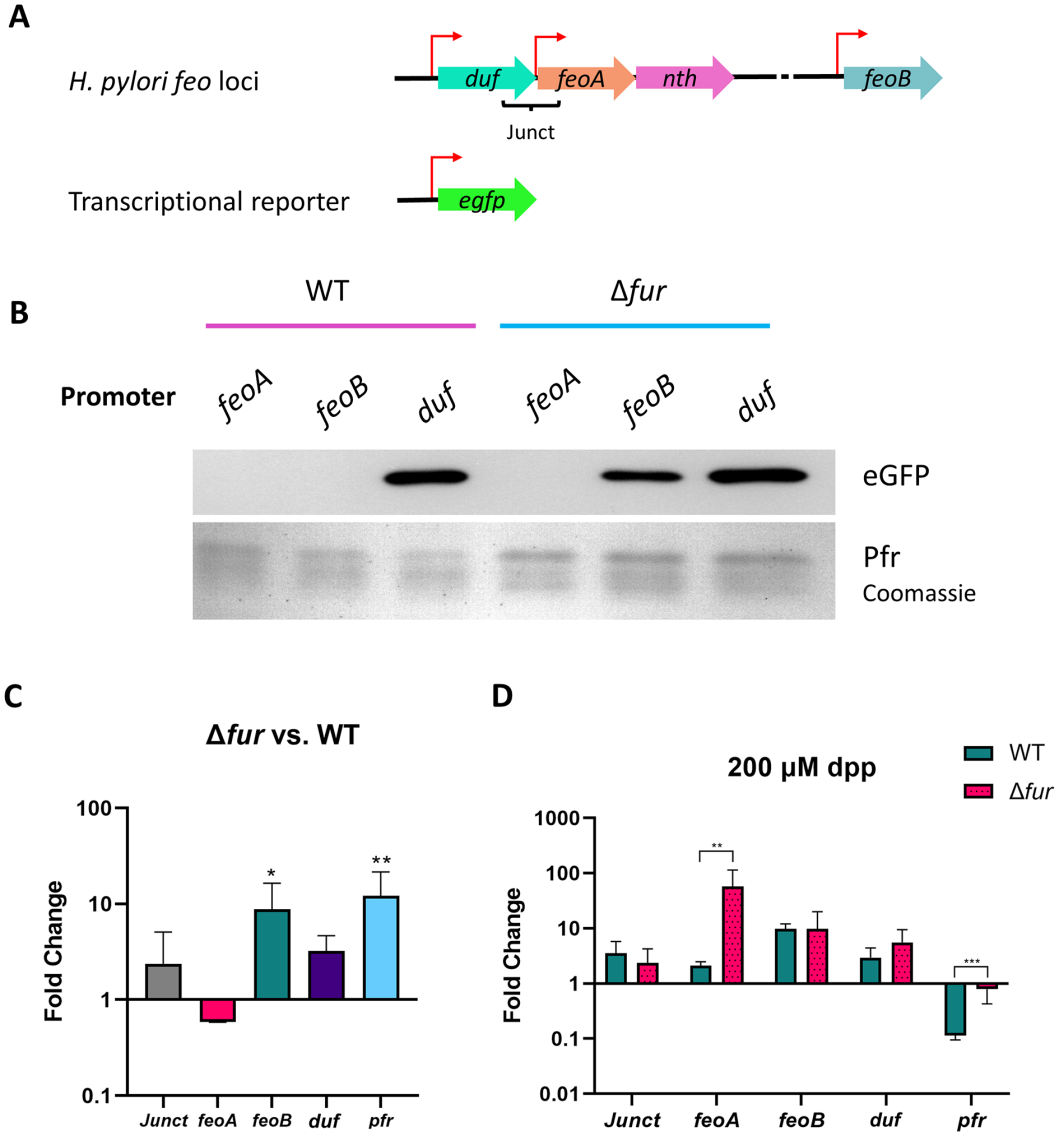
Deletion of the *duf-feoA-nth* transcription unit. **(A)** Three regions mapping the junctions between *duf*, *feoA*, and *nth* (labeled as A, B, and C) were amplified by PCR from cDNA produced from RNA of *H. pylori* G27. The expected size for each product is shown above the approximate location (scheme not to scale). **(B)** Results of PCR amplifications shown in A. gDNA refers to the positive controls for the amplification conditions using genomic DNA instead of cDNA. RT(−) corresponds to the negative controls for DNA contamination, where the reverse transcription was carried out in the absence of reverse transcriptase. Numbers on the left show the approximated size in bp as estimated from a DNA ladder.

To experimentally validate the existence of the *nth*-*feoA*-*duf* transcript, we used reverse-transcription PCR (RT-PCR) to amplify the junctions between these genes from mRNA of *H. pylori* (Figure 4). The RT-PCR tests yielded positive results for both gene junctions from the cDNA sample, confirming the existence of transcripts mapping across the junctions of these clustered genes. Notably, the existence of the *nth*-*feoA*-*duf* transcript is also supported by RNA-Seq data (Sharma et al., 2010). In sum, *in silico* and experimental evidence indicate that *nth*, *feoA*, and *duf* are co-transcribed in *H. pylori* G27, and likely comprise an operon.

### 3.3. *Hpfeoa* and *HpfeoB* are independently regulated, and only *HpfeoB* expression is directly modulated by Fur

**Figure 5:**
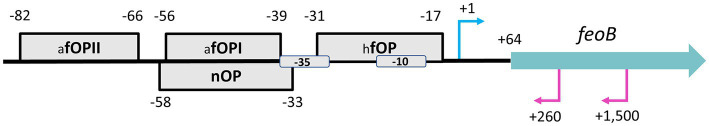
Activity of the putative *feo* promoters and gene transcription in *H. pylori* G27. **(A)** Schematic representation (not drawn to scale) of the transcriptional reporter used in these assays. Each putative promoter (depicted with red arrows) was amplified from *H. pylori* G27 genomic DNA and cloned upstream of a promoterless *gfp* gene in the pTM117 backbone. The junction between *duf* and *feoA* (Junct) is also shown. **(B)** Immunoblot analysis detecting GFP in *H. pylori* G27 strains, WT and Δ*fur*, transformed with the promoter fusions shown in panel A. Ferritin (Pfr), shown in the bottom lane from a Coomassie-stained gel, was used as a control of a Fur-regulated gene (upregulated in the absence of Fur). Both gels were loaded with the same samples. **(C)** Relative fold changes in the expression levels of the *HpfeoA*-*duf* junction (Junct), *HpfeoA*, *HpfeoB*, *duf*, and *pfr* in the Δ*fur* strain compared to the WT as determined by RT-qPCR. **(D)** Relative fold changes in gene expression in the Δ*fur* and the WT strains upon iron depletion induced with 200 μM dpp. The *p*-values for C and D were determined by an unpaired, two-tailed Student’s *t-*test from the ΔCT values. Differences that were statistically significant are indicated (*p < 0.05, **p < 0.01, ***p < 0.001). The bars correspond to the relative means and standard deviations (error bars) from four biological replicates. Statistical analyses and bar graphs were generated with GraphPad Prism v9.5.0.

Fur and FNR regulate the transcription of the *feo* operon in many species (Lau et al., 2016; Sestok et al., 2018). *H. pylori* lacks FNR, but encodes a Fur protein (Tomb et al., 1997). To examine the promoters controlling the expression of the *feo* genes in *H. pylori*, and a potential role of Fur in their regulation, we employed a *gfp*-based transcriptional reporter to test whether the sequences upstream of *feoA* and *feoB* have promoter activity (Figure 5A). Because *feoA* is the second gene of an operon, its transcription is likely controlled by the promoter of the first gene (i.e., *duf*). However, this does not rule out the possibility of *feoA* having its own, additional promoter. Thus, we included both the putative *duf* promoter and the sequence immediately upstream of *feoA* in our analyses. Similarly, we included the junction between both genes since this region should be present in the mRNA according to our prediction of the *duf*-*feoA*-*nth* transcription unit.

*Helicobacter pylori* G27 and a derivative Δ*fur* strain were transformed with the plasmids bearing the *gfp* fusions. Detection of Pfr (ferritin) was included as a positive control for a Fur-regulated product in these assays (Bereswill et al., 2000). The activity of the *duf* promoter (Figure 5B) did not depend on the presence of Fur, as this construct yielded high levels of GFP in both strains as determined via immunoblot analysis. In contrast, GFP was synthesized from the *feoB* promoter only in the Δ*fur* strain, indicating that this promoter is repressed by Fur in the WT background. Strikingly, GFP was synthesized in neither the WT nor the Δ*fur* background from the construct carrying the *feoA* promoter. This suggests that the *feoA* promoter might depend on a regulatory network not directly linked to Fur, and the experimental conditions we used were not adequate to turn on its expression. Consistent with these results, GFP synthesis from the three promoters did not show any changes upon dpp addition in the Δ*fur* strain (Supplementary Figure S2). Importantly, although the *feoA* promoter was not active under these conditions, transcripts encoding FeoA might still be present since the *duf* promoter is predicted to control the expression of the whole *duf*-*feoA*-*nth* unit. Thus, the *duf* promoter may work as the primary source of *feoA* transcription under certain conditions.

To further analyze how Fur affects the expression of the *feo* genes in *H. pylori*, we quantified the expression of the *feo* genes in both the WT and the Δ*fur* strains via RT-qPCR (Figure 5C). Only the expression of *feoB* was significantly different between the WT and the Δ*fur* backgrounds, with an average 10-fold increase in the absence of Fur. In contrast, changes in transcription levels for *feoA*, *duf*, and the junction between them were not significant. Taken together, the results of RT-qPCR assays agree with those obtained with the transcriptional reporter; *feoB* transcription is repressed by Fur in the WT strain, while *duf* and *feoA* do not appear to be directly regulated by Fur.

Because Fur is an iron-responsive transcription factor, we used RT-qPCR to assess how the expression of the *feo* genes in *H. pylori* responds to iron starvation, and whether such a response varies between the WT and the Δ*fur* strains. We induced iron starvation with 2,2′-dipyridyl (dpp), a Fe^2+^ chelator routinely used to study Fur-mediated regulation in *H. pylori* (Merrell et al., 2003; Carpenter et al., 2007, 2009; Pich et al., 2012). Our results (Figure 5D) showed that *pfr* had the expected response for a Fur-regulated gene, insofar as dpp addition resulted in a significant downregulation of transcription in the WT strain, but this response was absent in the Δ*fur* strain, indicating a direct Fur-dependent effect. Consistent with our previous results, changes of relative mRNA levels upon iron depletion did not vary significantly between the WT and the Δ*fur* backgrounds for *duf* and its junction with *feoA*. Strikingly, we did not observe any significant change in the expression of *feoB*, as would have been expected for a Fur-regulated gene. This may suggest that our experimental conditions (i.e., 200 μM dpp over 2 h) were not enough to induce a measurable response in the *feoB* transcription levels; and, as discussed in detail below, the regulation of *feoB* by Fur is predicted to be more complex in *H. pylori* than in other species. Finally, *feoA* was upregulated in conditions of iron starvation but only in the Δ*fur* strain; hence, this response might not be directly related to Fur, and, as anticipated from our immunoblot analysis, additional layers of regulation could be involved in the expression of this gene.

When transformed into a *fur*-null strain of *E. coli*, all three promoter-*gfp* fusions led to the synthesis of GFP (Supplementary Figure S3). Altogether, these results indicate that the sequences upstream of *feoA*, *duf*, and *feoB* are promoters of different strengths (reflected in the production of different GFP amounts under the same conditions); and transcription of *HpfeoA* relies on two promoters, that of the *duf*-*feoA*-*nth* operon; and that of *HpfeoA*, which might serve as a promoter for a *feoA*-*nth* suboperon.

### 3.4. Sequence analysis suggests that Fur and NikR govern transcription of *HpfeoB*

**Figure 6:**
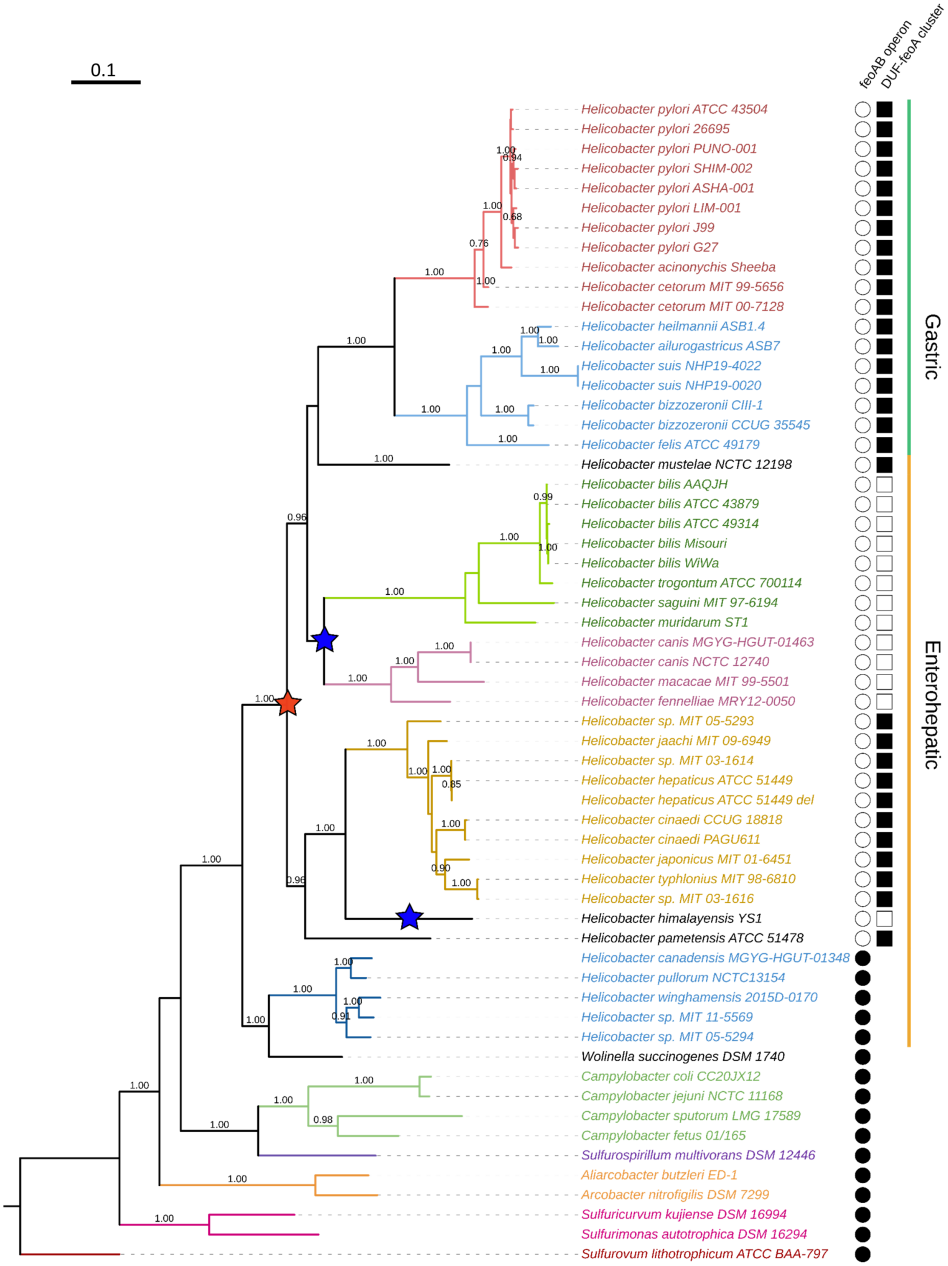
Schematic representation of the proposed *HpfeoB* promoter architecture. The gray boxes represent the identified operator sites for apo-Fur (afOP I and II), holo-Fur (hfOP), and NikR (nOP) with their relative positions indicated by the numbers around each box. All binding motifs were found in the same direction of *HpfeoB*, and their location in this scheme, either on the top or the bottom, is only for illustrative purposes. Proposed −10 and − 35 boxes are also shown. The transcription start site of *HpfeoB* (position +1) and the anti-sense RNAs (+260 and + 1,500) identified by Sharma et al. (2010) via RNA-Seq are depicted with the cyan (above the line) and magenta (below the line) arrows, respectively. The thick blue arrow represents the position and directionality of the *HpfeoB* coding sequence. This model is based on the *H. pylori* G27 genome and is not drawn to scale.

To identify those transcription factors regulating the expression of the *feo* genes in *H. pylori*, including Fur, we searched for conserved binding motifs in the putative *feo* promoters using XSTREME (Grant and Bailey, 2021), a pipeline based on Hidden Markov Models (HMM). XSTREME identifies enriched motifs in aligned sequences, and compares them with those deposited in reference databases or provided by the user to find significant matches. We found that the *H. pylori feoB* promoter has conserved, overlapping binding boxes for the apo (dimeric, Fe^2+^-free) and holo (tetrameric, Fe^2+^-bound) forms of Fur (Figure 6 and Supplementary Figure S4), which are both active in *H. pylori* (Agriesti et al., 2014). Our analysis also identified binding sequences for the Ni^2+^-responsive transcription factor NikR. In addition, a previous study of the primary transcriptome of *H. pylori* identified at least two small anti-sense RNAs encoded within the *feoB* coding sequence (Sharma et al., 2010). Therefore, the expression of *feoB* in *H. pylori* is likely modulated by the presence of iron and nickel through a complex interplay of Fur and NikR and, potentially, small anti-sense RNAs.

**Figure 7:**
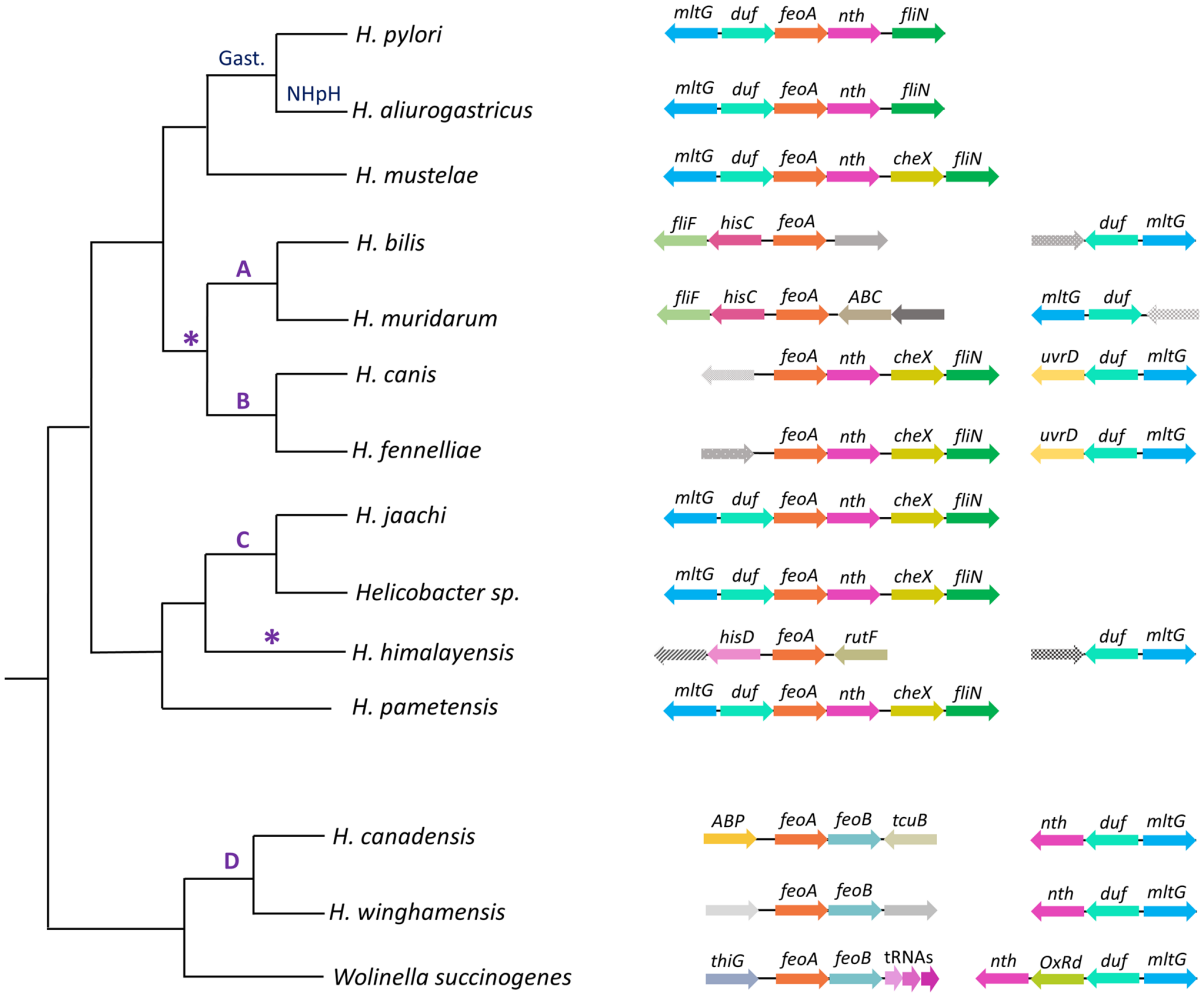
Phylogenetic reconstruction of the Campylobacterota group. This tree was constructed by the maximum likelihood method based on the alignment of concatenated RpoB-RpoC protein sequences. Bootstrap values were calculated with 300 replicates, and those values over 0.5 are shown on the corresponding nodes. Colors indicate clustering according to the currently proposed classification of the helicobacters. From top to bottom: *H. pylori* group (red), non-*H. pylori* gastric helicobacters (light blue); enterohepatic helicobacters groups A, B, C, and D (dark green, magenta, gold, and blue, respectively). The ones in black are not classified within these groups. Non-*Helicobacter* species are shown in the bottom and colored according to their genus affiliation: from *Campylobacter* spp. to *Sulfurovum lithotrophicum* (outgroup).

When examining the putative promoters of *duf* and *feoA*, we did not find any significant match for Fur or NikR binding sequences, nor for any other transcription factors. Thus, there is no evidence of direct Fur-dependent regulation on these genes. This supports our previous findings that there is not a single regulatory network controlling both *feoA* and *feoB* in *H. pylori*. In Figure 7, the first column on the right shows whether the *feo* operon architecture is conserved (filled in black) or not (white) in each species. Similarly, the second column shows the presence (filled black square) or absence (white) of the *duf-feoA* cluster. The red star indicates the node in which we hypothesize the initial operon split occurred, and the blue stars indicate the later *feoA* rearrangement events proposed in our model.

### 3.5. The split of the *feo* operon occurred after the divergence of the family Helicobacteraceae

To determine how *H. pylori* evolved its unique architecture of the *feo* genes and whether this is a feature exclusive to this species, we carried out a phylogenetic reconstruction of the Campylobacterota [Epsilonproteobacteria] group to trace back the relative position of the *feo* loci. Our tree (Figure 7) was constructed based on the β and β’ unit of the RNA-polymerase (RpoBC), and recapitulated the current taxonomy of this group (Solnick and Vandamme, 2001; Dewhirst et al., 2005; Waite et al., 2017; Smet et al., 2018; Prada et al., 2022), insofar as the *Helicobacter* genus forms a monophyletic group together with *Wolinella succinogenes*. Within this group, the gastric helicobacters form a monophyletic group containing the clades of the *H. pylori* and the non-*H. pylori* helicobacters. The enterohepatic helicobacters formed a paraphyletic group made up of several discrete clades that other authors have proposed as genera with pending nomenclature, temporarily referred to as *Helicobacter* A, B, C, and D (Waite et al., 2017). In this text, we follow this temporary nomenclature as suggested by Waite and colleagues.

According to our phylogenetic tree, the split of the *feo* operon has a single evolutionary origin that dates to the common ancestor of most—but not all—the current helicobacters, excluding the *Helicobacter* D group. By contrast, our tree does not show a single node that differentiates those species with the *duf-feoA* cluster from those with other arrangements. Instead, the most parsimonious scenario given our tree is that in which the split of the ancestral *feo* operon correlated with the emergence of the *duf-feoA* cluster, but at least two independent additional rearrangements involving *feoA* took place later in the evolution of certain helicobacters: Namely, in *H. himalayensis* and the common ancestor of *Helicobacter* A and B. These latter groups are likely to comprise separate genera within the current *Helicobacter* classification, and are represented in our tree (Figure 7) by *H. bilis*, *H. trogontum*, *H. saguini*, and *H. muridarum* (*Helicobacter* A); and *H. canis*, *H. macacae*, *and H. fennelliae* (*Helicobacter* B).

**Figure 8:**
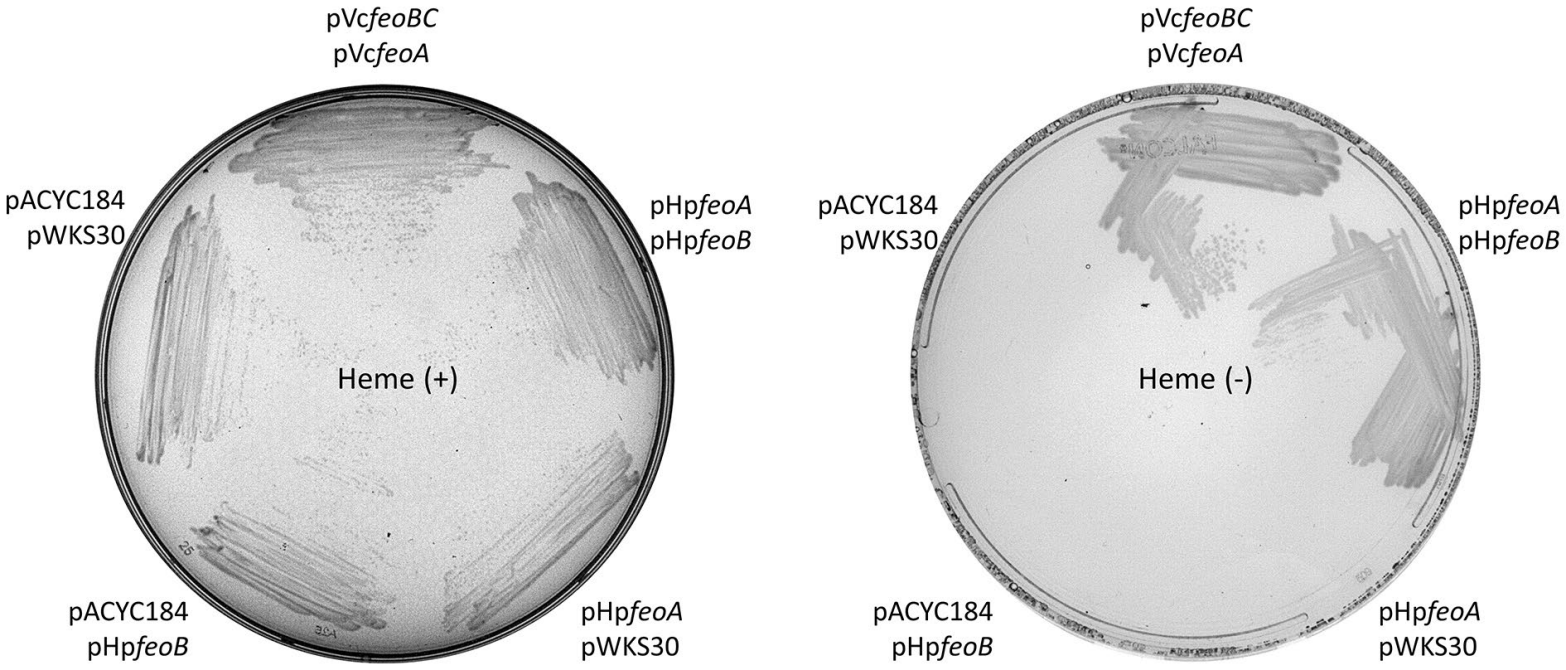
Genomic context of *feoA* loci among *Helicobacter* species. This cladogram shows the phylogenetic relationship as found in the organismal tree above for representative species of the gastric (Gast.) and non-*H. pylori* helicobacters (NHpH) as well as for the groups A, B, C, and D of the enterohepatic helicobacters (as indicated above the corresponding node). The AB node and the *H. himalayensis* branch, in which additional *feoA* rearrangements might have taken place, are indicated with an asterisk (*). The genomic context of the *feoA* and *duf* loci are shown after each species following the gene annotation deposited in GeneBank for each genome. Distances and gene sizes are not drawn to scale.

The synteny of the *feoA* locus across the helicobacters (Figure 8) shows that both the *feo* operon and the association between the *nth*, *duf*, and *mltG* genes are conserved in the *Helicobacter* D group and *W. succinogenes;* while the *duf*-*feoA*-*nth* arrangement is prevalent in most of the helicobacters lacking the operon structure (i.e., all but the D group). This finding is consistent with the hypothesis of an ancestral *feo* operon that split upon the divergence of the ancestor shared by the gastric helicobacters and the groups A, B, and C. *H. himalayensis* as well as *Helicobacter* A and B do not share synteny of the *feoA* locus, suggesting that these groups underwent independent rearrangements of *feoA* after the primary split event of the ancestral operon. Since the species of group B conserves the order *feoA*, *nth*, *cheX*, *fliN* observed in other groups, we propose as the most parsimonious scenario is that group A evolved a second relocation of *feoA*, albeit alternative models are plausible.

Considering the probable ancestral operon structure of *feo* as well as our promoter analyses showing that Fur and NikR likely regulate the expression of *HpfeoB* but not *HpfeoA*, we sought to determine whether the nucleotide sequences underlying this regulation existed in the ancestral operon and were conserved only in *feoB* upon operon excision. To investigate the feasibility of this hypothesis, we applied the same HMM-based approach described above to look for conserved transcription factor binding motifs in promoters of the *feo* operon in *Helicobacter* D and other Campylobacterota species. Indeed, this analysis showed that the putative promoter of the *feo* operon has a distribution of potential binding sequences for Fur and NikR similar to that of the *feoB* promoter (Supplementary Figure S5).

### 3.6. HpFeoA and HpFeoB interact to form a transmembrane complex

The current mechanistic model of the Feo system posits that FeoA, FeoB, and FeoC interact to assemble a large complex embedded in the inner membrane (Stevenson et al., 2016). We used a vector encoding a C-terminal FLAG-tagged version of *H. pylori* FeoA (HpFeoA^C-FLAG^) to test whether this protein forms a membrane complex in association with HpFeoB in EPV6. Expression *in trans* of *HpfeoA*^C-FLAG^ and *HpfeoB* supports EPV6 growth in non-supplemented LB agar, the same as co-expression of the *HpfeoB* together with *HpfeoA* (without the epitope tag), indicating that the C-terminal FLAG tag in HpFeoA does not affect its function.

**Figure 9:**
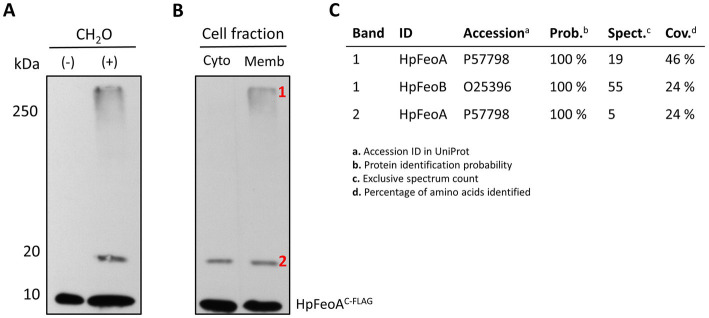
Formation of the transmembrane Feo complex by HpFeoA and HpFeoB in *V. cholerae* EPV6. **(A)** Immunoblot analysis of *V. cholerae* EPV6 co-transformed with plasmids carrying HpFeoA^C-FLAG^ and HpFeoB. CH_2_O indicates whole cell pellets before (−) and after (+) formaldehyde crosslinking *in vivo*. **(B)** Samples obtained upon cell fractionation: cytoplasmic and membrane fractions (labeled as Cyto and Memb, respectively.) The numbers on the left indicate the estimated protein size in kDa. **(C)** Results for peptides matching HpFeoA and HpFeoB retrieved from mass spectroscopy analysis of those bands in the membrane fraction (labeled as 1 and 2) after immunoprecipitation with a monoclonal anti-FLAG antibody.

EPV6 cells transformed with plasmids harboring *HpfeoA*^C-FLAG^ and *HpfeoB* were crosslinked *in vivo* with formaldehyde and then visualized via immunoblot analysis to identify complex formation. In addition to the HpFeoA^C-FLAG^ monomer, there were two additional bands reactive to the anti-FLAG antibody that appeared upon crosslinking (Figure 9A). The approximate sizes of the bands are consistent with a HpFeoA^C-FLAG^ dimer (20 kDa) and the large transmembrane Feo complex (250 kDa), as observed in the *V. cholerae* model (Stevenson et al., 2016).

To determine whether the >250 kDa band corresponds to a transmembrane complex, we carried out cell fractionation via ultracentrifugation on the crosslinked sample. Immunoblot analysis of the cell fractions revealed that the large complex was indeed present only in the membrane fraction (Figure 9B). Similarly, to confirm the presence HpFeoA^C-FLAG^ in both the 20 kDa and the >250 kDa bands, we enriched the crosslinked sample through immunoprecipitation using a monoclonal anti-FLAG antibody, excised the corresponding bands from the acrylamide gel, and analyzed them via mass spectrometry. Peptides mapping to HpFeoA were found in both samples, and peptides corresponding to HpFeoB were also identified in the >250 kDa band (Figure 9C). Together, these findings suggest that HpFeoA forms a dimer, and that HpFeoA and HpFeoB interact to form a complex in the cytoplasmic membrane similar to that observed in *V. cholerae* (Stevenson et al., 2016; Gómez-Garzón and Payne, 2020).

Attempts to validate these results with tagged HpFeoA or HpFeoB proteins in *H. pylori* were unsuccessful. The tagged proteins either were not detected or interfered with function.

**Figure 10:**
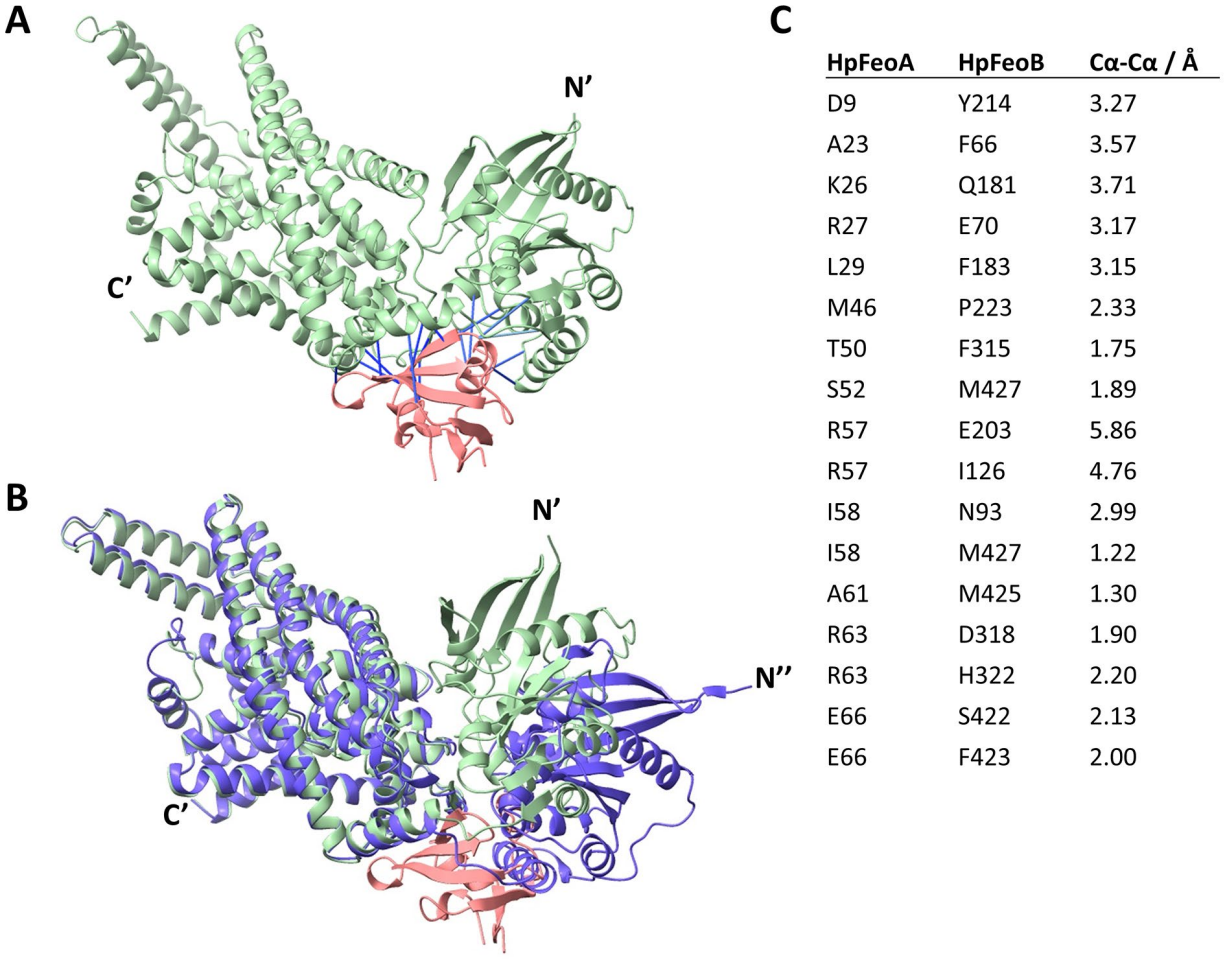
Structural model of the HpFeoA-HpFeoB interaction constructed with AlphaFold-Multimer. **(A)** 3D representation of the best model obtained for the interaction between HpFeoA (WP_000174130, in pink) and HpFeoB (WP_041201363, in green) by AlphaFold-Multimer. N′ and C′ correspond to the N- and C- termini of HpFeoB. The blue lines represent the predicted contacts between the two proteins, defined as residues at 3.00 Å or closer to each other. These contacts have a predicted aligned error value of 0. **(B)** Structural alignment of the HpFeoA-HpFeoB model shown in panel A and the 3D model for HpFeoB alone deposited in AlphaFold DB (ID: B5Z754_HELPG, shown in purple). N″ represent the N-terminus of the latter model as it does not align with that of the interaction model. **(C)** Predicted interacting peptides between HpFeoA and HpFeoB shown in **(A)**, indicating the predicted distance between alpha carbon (Cα-Cα).

Finally, we modeled the potential HpFeoA-HpFeoB interaction *in silico* through AlphaFold-Multimer (Evans et al., 2022), a recently released extension of the AlphaFold deep learning pipeline that allows to model protein–protein interactions from amino acid sequences. As shown in Figure 10A, our model predicts interacting residues in HpFeoB mapping to transmembrane, as well as the N-terminal, domains of the protein, which contrasts with the long-standing model in which FeoA interacts only with the N-terminal domain of FeoB. Our model also predicts that HpFeoB has a hinge close to the interface between the N-terminal and the transmembrane domains, about Ala208, which undergoes an important change upon HpFeoA binding. Namely, when compared the HpFeoA-bound form of HpFeoB to that of the protein by itself, our model shows that HpFeoA stabilizes a specific conformation of HpFeoB, by locking this hinge in place (Figure 10B and Supplementary Figure S6). This suggests that HpFeoA might trigger structural changes in HpFeoB, which could indicate a regulatory role. We identified those amino acids located in the interacting surfaces of both proteins (Figure 10C). These residues are likely to have a functional significance for the HpFeoA-HpFeoB interaction and thus they may be useful targets in further mutational analyses.

## 4. Discussion

*Helicobacter pylori* is a widespread human commensal that colonizes the gastric mucosa. Chronic infection with *H. pylori* triggers an inflammatory response that, in some cases, may lead to the development of metaplasia and, ultimately, cancer. This bacterium is indeed considered the major risk factor for the development of peptic ulcers and gastric cancer worldwide (Herrera and Parsonnet, 2009; Plummer et al., 2015; de Martel et al., 2020). Therefore, *H. pylori* is an important human pathogen, and efforts to advance our understanding of this pathogen must be a priority.

By studying the evolutionary history of the bacterial ferrous iron transporter, Feo, we found the unique *H. pylori* gene architecture for this system. There are two *feo* genes in the *H. pylori* genome, *feoA* and *feoB* but, unlike in most species, they are not clustered in an operon, but separated by 116 kbp. This architecture is conserved among most helicobacters. We examined the functional implications of this arrangement by evaluating the requirement of *HpfeoA* and how *HpfeoA* and *HpfeoB* are regulated. We found that, although *HpfeoA* is encoded in an operon together with two other unrelated genes (*nth* and *duf*), it is needed for the function of the Feo transporter, demonstrating that *H. pylori* Feo is a two-component system encoded by distant loci.

The Feo system is widespread among bacteria, and although the *feo* genes are clustered in species other than *Helicobacter*, there is diversity in their number and organization (Lau et al., 2016; Sestok et al., 2018; Gómez-Garzón et al., 2022). For example, some species have two *feoA* genes, though whether both genes are needed or have different roles is yet to be determined. Also, it is common within the Firmicutes and Bacteroidetes that Feo is made up of a single protein containing a fusion of FeoA- and FeoB-like domains (Gómez-Garzón et al., 2022). A study in *Rhodobacter capsulatus* found that this bacterium has two *feo* gene clusters, but only one conserves iron transport activity, while the other one functions as a manganese transporter (Zappa and Bauer, 2013), showing that a duplication event may have led to the evolution of a homolog transporter. These studies suggest that gene rearrangements have shaped the evolution of Feo, and hence *feo* gene architecture may correlate with different mechanistic features. It was plausible, for example, that *H. pylori* dispensed with *feoA,* so that this gene has evolved separately and probably acquired a new function in the context of the operon it makes with *nth* and *duf*. However, our data ruled out this scenario, showing that both *feoA* and *feoB* are necessary and sufficient for iron transport, and deletion of either gene results in a similar phenotype (Figures 3, 4).

We propose that the split of the *feo* operon occurred early in the evolution of the Campylobacterota group, before the divergence of most Helicobacteriaceae genera, excluding only the ancestor of the species of the *Helicobacter* D group. We also hypothesize that this split resulted in the association of *feoA* and *duf*, but additional rearrangements of *feoA* took place later in the ancestors of the groups A and B (Figure 2). Our analyses indicate that the ancestral *feo* operon was likely regulated by Fur and NikR but only *feoB* conserved the ancestral promoter upon operon splitting, while *feoA* became dependent on the new operons it formed. Two questions emerge from this evolutionary scenario. First, how did *HpfeoA* acquire its own promoter? The current *HpfeoA* promoter may have retained key features of the ancestral promoter, but did not retain NikR/Fur regulation. Equally possible is that *HpfeoA* evolved a promoter *de novo*; it has been shown that random mutations can lead to the evolution of promoters (Yona et al., 2018). Second, has the split of the *feo* operon been positively selected in *Helicobacter* spp., i.e., how does the split of the *feo* operon affect the fitness of these species to thrive in the host environment? Since *H. pylori* is characterized by its highly plastic genome (Blaser and Berg, 2001; Suerbaum and Josenhans, 2007), and it is considered to comprise a panmictic population (Salaün et al., 1998), those factors preserving the split *feo* gene architecture in this group merit further study.

Little was known about *HpfeoA* and its role in iron uptake because the annotation of the first genome of *H. pylori* (Tomb et al., 1997) did not include this gene. This was likely due to its small size (230 bp) and location between two other unrelated genes, which made it difficult to be recognized by the annotation pipelines used at the time. In consequence, it has been largely assumed that *H. pylori* Feo relied solely on *feoB*. More recent genome annotations have identified *feoA* homologs throughout all the Campylobacterota group, and Müller et al., 2013 demonstrated through proteomics analysis that *H. pylori* 26,695 synthesizes the FeoA protein. Velayudhan and colleagues (Velayudhan et al., 2000) showed, using a mouse model, that *feoB* is a major contributor for virulence in *H. pylori* 4187E. Future studies will determine whether Feo is a major determinant for virulence in other clinically relevant *H. pylori* strains, and whether our findings regarding the requirement of *feoA* can be expanded to these models.

*Helicobacter pylori* has closely coevolved with humans for more than 100,000 years (Moodley et al., 2012). The genome of this species exhibits an unusually high plasticity and has been largely shaped by horizontal gene transfer and recombination events (Garcia-Vallvé et al., 2002; Gressmann et al., 2005; Prada et al., 2022). It is not unexpected then that *H. pylori* lacks several operon arrangements and master regulators widely conserved among bacteria. Among the missing transcription factors in *H. pylori* is FNR, which, in Gammaproteobacteria, controls the expression of the *feo* operon in response to changes in oxygen tension. We found that the transcription of *feoB* in *H. pylori* is likely to be tightly regulated by Fur and NikR, hence *H. pylori* controls the expression of *feoB* depending on the availability of iron and nickel. Fur and NikR have been shown to form an intricate regulatory network in *H. pylori* (Delany et al., 2005; Danielli et al., 2010; Roncarati et al., 2016; Vannini et al., 2022). These transcription factors co-regulate genes essential for cell homeostasis, such as the *exbB-exbD-tonB* operon, which encodes a complex that provides energy to several ATP-driven transporters. In addition, Fur and NikR regulate the transcription of one another; namely, holo-NikR represses fur expression and holo-Fur represses NikR expression. Fur also impacts *NikR* expression as well as NikR-regulated genes and vice versa. The NikR and Fur regulons include important virulence factors and central metabolism genes (Vannini et al., 2022); for instance, NikR regulates the expression of the urease, essential for the colonization of the gastric mucosa; and Fur modulates the expression of *cagA* (involved in inflammatory response), *pfr* (ferritin), *arsRS* (master regulator), and *amiE* (amidase, critical in nitrogen metabolism), among others (Delany et al., 2005; Danielli et al., 2010; Roncarati et al., 2016).

Fur-mediated regulation is particularly complex in *H. pylori*. In addition to the interplay with NikR described above, both the holo (dimeric) and apo (monomeric) forms of Fur are active and bind different sequences in the DNA (Agriesti et al., 2014). Therefore, regulation by Fur is often the result of a competition between the two forms of the protein, on top of additional kinetic factors involving oligomerization as well as iron and DNA binding. This means that *H. pylori* Fur works like a commutator switch rather than like a simple ON/OFF switch. This feature, not reported in other species, likely evolved in *H. pylori* as a means to overcome the absence of other transcription factors (Agriesti et al., 2014). Based on sequence analysis, we anticipate that NikR and both forms of Fur are involved in the regulation of *HpfeoB* (Figure 7). In addition, a comprehensive RNA-Seq study on the primary transcriptome of this pathogen (Sharma et al., 2010) identified two small anti-sense RNAs encoded within the *feoB* coding sequences, which may form an additional layer of regulation.

We found that *feoA* transcripts are produced from two promoters (Figure 6), the promoter for *duf*—which also involves the *nth* gene and seems to be constitutively expressed—and an internal promoter. Interestingly, we found no evidence of Fur- or NikR-mediated regulation on *feoA*. Considering the stringent regulation to which *feoB* is subjected, we hypothesize that the regulation of the Feo system in *H. pylori* primarily relies on controlling *feoB* expression, while *feoA* transcripts may be readily available in the cell. Thus, *feoB* mRNA abundance might serve as a bottleneck for the assembly of the Feo transporter.

We used dpp-induced iron starvation to study regulation by Fur in *H. pylori* as reported in previous studies (Carpenter et al., 2007). These conditions led to significant repression of the *pfr* gene (Figure 6), indicating they serve to assess Fur-mediated response. However, dpp can bind metal cations other than iron, and Fur has been found responsive to these ions as well (Bereswill et al., 2000); thus, our results might reflect the effects of changes in iron availability along with other metals. The effects of copper on *H. pylori* gene regulation have been determined (Waidner et al., 2002), and neither the *feoA* nor the *feoB* operons were shown to be regulated by copper. Future studies should test additional metals, especially nickel, to determine whether any metals other than iron govern the expression of *feoB* in *H. pylori*.

While this study provides initial insight into Feo-mediated iron transport in *H. pylori*, those environmental conditions (i.e., changes in Fe^2+^ and Ni^2+^ concentration) necessary to induce a consistent response in *HpfeoB* and *HpfeoA* need further characterization. We did not find a specific set of experimental conditions in which the *feoA* promoter was up-regulated using our transcriptional reporter. Likewise, we did not observe a switch in *feoB* transcription in response to Fur in our RT-qPCR assay, though all our other analyses and the scientific literature show that Fur regulates this gene. We anticipate a high level of complexity in the regulation of these genes. Fur and NikR regulation on *feoB* and the ancestral *feo* operon are based on sequence analyses; hence, experimental validation, including evidence of physical protein-DNA interaction and effects of sequence variability (i.e., mismatches from the consensus sequence) on binding affinity, is needed to fully elucidate the role of these master regulators in modulating the expression of *feoB*.

In previous studies conducted in *V. cholerae*, we have found that FeoA, B, and C assemble a multimeric complex in the inner membrane (Stevenson et al., 2016), likely composed by trimers of FeoABC units, and that FeoB may form intermediate oligomers with FeoA or by itself before assembling the large complex. Consistent with these findings, some authors have observed that purified NFeoB or full-length FeoB forms trimers *in vitro* (Guilfoyle et al., 2009; Hagelueken et al., 2016; Seyedmohammad et al., 2016), although little is known about the relevance of these complexes *in vivo*. Here, we found that HpFeoA and HpFeoB form a membrane complex when expressed in *V. cholerae*, and that HpFeoA also formed dimers (Figure 9). However, a limitation of this study was that we were unable to assess Feo complex formation directly in *H. pylori*.

3D structural modeling of the HpFeoA-HpFeoB interaction using AlphaFold-Multimer (Evans et al., 2022) predicts an area of structural flexibility in HpFeoB that undergoes a structural shift when interacting with HpFeoA (Figure 10 and Supplementary Figure S6). This suggests that HpFeoA might interact with HpFeoB to trigger a regulatory response for either complex formation or iron uptake. Although it has been suggested that FeoA could regulate the NTPase activity of FeoB, FeoA does not affect the enzymatic activity of full-length FeoB (Lau et al., 2013; Gómez-Garzón and Payne, 2020). Therefore, we propose that structural changes in FeoB induced by FeoA may be involved in regulating pore opening or the assembly of the large complex. Our model yielded high confidence structures (Supplementary Figure S7); and thus, the putative interacting amino acids we identified should guide further studies seeking to discern the functional significance of this protein–protein interaction. Membrane-associated proteins, such as FeoB, present a major hurdle for both crystallographic analysis and *in silico* modeling; thus, mutational analysis is an important approach to fully characterize the interaction between HpFeoA and HpFeoB, especially in the absence of full-length crystal structures of FeoB. Also, there are some factors missing in the AlphaFold-Multimer model that may have an effect *in vivo*. For instance, the presence of an NTP molecule bound to HpFeoB may induce additional changes in this protein; oligomerization of HpFeoB as well as membrane localization may significantly change the way in which HpFeoB interacts with HpFeoA.

In summary, our studies identified the remarkable differences between the *H. pylori* Feo system and those of the Gammaproteobacteria group, which have largely been used as model organisms. Establishing the *H. pylori* Feo as a new model for Feo will represent a leap toward a more comprehensive understanding of this important bacterial transporter, especially in the context of bacterial pathogens.

## Data availability statement

The original contributions presented in the study are included in the article/Supplementary material, further inquiries can be directed to the corresponding author.

## Author contributions

CG-G and SP contributed to conception and design of the study. CG-G carried out the experimental procedures, statistical analyses, and wrote the first draft of the manuscript. SP contributed to the analysis of the data and experimental design. All authors contributed to manuscript revision, read, and approved the submitted version.

## Funding

This work was funded by the National Institutes of Health (NIH) (grant R01 AI091957) to SP.

## Conflict of interest

The authors declare that the research was conducted in the absence of any commercial or financial relationships that could be construed as a potential conflict of interest.

## Publisher’s note

All claims expressed in this article are solely those of the authors and do not necessarily represent those of their affiliated organizations, or those of the publisher, the editors and the reviewers. Any product that may be evaluated in this article, or claim that may be made by its manufacturer, is not guaranteed or endorsed by the publisher.
