# Supplementary material for: Divide and conquer: genetics, mechanism, and evolution of the ferrous iron transporter Feo in Helicobacter pylori
Source: Front Microbiol. 2023 Jul 4;14:1219359. doi: 10.3389/fmicb.2023.1219359 (PMC10353542; doi:10.3389/fmicb.2023.1219359)
Supplement: Supplementary file 1 [file Data_Sheet_1.pdf]

Supplementary Material

Divide and Conquer: Genetics, Mechanism, and Evolution of the Ferrous Iron Transporter Feo in *Helicobacter pylori*

Camilo Gómez-Garzón[1,#], Shelley M. Payne[1,2 *]

[1]Department of Molecular Biosciences, University of Texas at Austin, Austin, TX, United States.

[2]John Ring LaMontagne Center for Infectious Disease, The University of Texas at Austin, Austin, TX, United States.

[#] Present address: Human Biology Division, Fred Hutchinson Cancer Center, Seattle, WA, United States.

*** Correspondence:**
Shelley M. Payne
payne@utexas.edu

Supplementary Table 1. Bacterial strains used in this study

Strain	Description	Reference or source
E. coli		
TOP10	F- *mcrA* Δ(*mrr-hsdRMS-mcrBC*) φ80*lacZ*ΔM15 Δ*lacX*74 *nupG* *recA*1 *araD*139 Δ(*ara-leu*)7697 *galE*15 *galK*16 *rpsL*(*strR*) *endA*1 λ-	Invitrogen
H1771	MC4100 *aroB feoB*7 *fhuF*::λplac Mu	Hantke 1987
MFT-5	*fur*::Tn5Kmr	M. McIntosh
V. cholerae		
EPV6	O395 Δ*vibB feoABC*::*kan vctP*::gent *fbpA*::cam	Peng 2016
H. pylori		
G27	WT *H. pylori*	Covacci 1993
Δ*fur* (DSM300)	G27 Δ*fur::cat*, Cmr	S. Merrell
Δ*feoA*	G27 Δ*feoA::cat*, Cmr	This study
Δ*feoB*	G27 Δ*feoB::cat*, Cmr	This study

Supplementary Table 2. Plasmids used in this study

Plasmid	Description	Reference or source
pACYC184	Medium-low copy cloning vector, CamR, TetR.	Chang 1978
pWKS30	Low-copy cloning vector, AmpR	Rong Fu 1991
pGTXN3	Cloning vector harboring a promoterless *gfpmut3* gene	Runyen-Janecky 1999
pTM117	*H. pylori* cloning vector harboring a promoterless *gfpmut3* gene	Merrell
pVcfeoBC (pFeoΔA)	pWKS30 carrying a in-frame Δ*feoA* mutant of the *V. cholerae feo* operon	Weaver 2013
pVcfeoA (pACfeoA)	pACYC184 carrying *V. cholerae feoA*	Weaver 2013
pHpfeoA	pACYC184 carrying *H. pylori feoA* controlled by the Tet promoter	This study
pHpfeoB	pWKS30 carrying *H. pylori feoB*	This study
pHpfeoA$^{C\text{-}FLAG}$	pHpfeoA with a C-terminal tag in *HpfeoA*	This study
pTMHpfeoA	*HpfeoA*::pTM117 complementation vector	This study
pGT-PHpfeoA	pGTXN3 carrying *HpfeoA* promoter::*gfpmut3* fusion	This study
pGT-PHpfeoB	pGTXN3 carrying *HpfeoB* promoter::*gfpmut3* fusion	This study
pGT-PHpduf	pGTXN3 carrying *Hpduf* promoter::*gfpmut3* fusion	This study
pTM-PHpfeoA	pTM117 carrying *HpfeoA* promoter::*gfpmut3* fusion	This study
pTM-PHpfeoB	pTM117 carrying *HpfeoB* promoter::*gfpmut3* fusion	This study
pTM-Phpduf	pTM117 carrying *Hpduf* promoter::*gfpmut3* fusion	This study

Supplementary Table 3. Primers used in this study

Primer name[a]	Sequence (5' – 3')
HpFeoA-PstC-R	AGCGTGCTGCAGTGAAAATGGGTTTAAAACGCGCTAAAACTCAC
C-FLAG-PstI-Top	GGACTACAAAGACGATGACGACAAGTAACTGCA
C-FLAG-PstI-Btm	GTTACTTGTCGTCATCGTCTTTGTAGTCCTGCA
promHpFeoA-XmaI-F	ATACCCGGGTGTCTTAAATAAAATCCCTATTGTGGGCTATC
promHpFeoA-BamHI-R	CTAGGATCCTTTACTTCCTTTTTGATTCAATGTTTTTTTTGACTTC
promHpFeoB-XmaI-F	ATACCCGGGGCTTTTCAAAACTCCATTCACAGATTTTTAGC
promHpFeoB-BamHI-R	CATGGATCCTGGGTTTGAATGCTCCGCATTAAAG
prom-DUF-XmaI-F	GTACCCGGGGCTCAAAATTTGCGTGAAAAAATAG
prom-DUF-XbaI-R	CGATCTAGATAGTATTCTTTTTTATTTAAGTATACCAATTTATCC
feoA-nth-F	GTTTTTATGGTGCAATTCGGTGG
feoA-nth-R	CCACGCTTTCGGTTAAAATCAACC
Duf-feoA-Locus-F	AGCGTGGCTTTTTTCATGGAATAATGC
Duf-feoA-Locus-R	AAATCCCTATTGTGGGCTATCTTGTC
RTq-junct-F	AGGGCTTCATCGCAGTTAGC
RTq-junct-R	AATCTTGCGCCGTATTTTCAC
RTq-HpfeoA-F	ACTGCGATGAAGCCCTTAAA
RTq-HpfeoA-R	GCGGTTGATTTTAACCGAAA
RTq-pfr-F	GGGCTTTTCTTGTTTGACCA
RTq-pfr-R	TTGCACAGGCACATTGTTTT
RTq-HpfeoB-F	TCATCAACGCTTTGAGCAAC
RTq-HpfeoB-R	ATGGATCAAACCCACTTCCA
RTq-duf-F	AAGTCCAAGTGGATGGCAAC
RTq-duf-R	CGGTTAGCCCTTGCAAATAA
RTq-gyrB-F	CGCCCTGGAATGTATATTGG
RTq-gyrB-R	CAGCGTTATCCACGACTTCA
RTq-rpoD-F	GAATTGTTCGCCAATTCCTT
RTq-rpoD-R	GGGCTAGAGTGGGGACTTTC

[a] When present, restriction sites included in the primer are underlined in the primer's name.
