# Supplementary material for: Divide and conquer: genetics, mechanism, and evolution of the ferrous iron transporter Feo in Helicobacter pylori
Source: Front Microbiol. 2023 Jul 4;14:1219359. doi: 10.3389/fmicb.2023.1219359 (PMC10353542; doi:10.3389/fmicb.2023.1219359)
Supplement: Supplementary file 2 [file Data_Sheet_2.pdf]

Supplementary Material

Divide and Conquer: Genetics, Mechanism, and Evolution of the Ferrous Iron Transporter Feo in *Helicobacter pylori*

Camilo Gómez-Garzón[1,#], Shelley M. Payne[1,2 *]

[1]Department of Molecular Biosciences, University of Texas at Austin, Austin, TX, United States.

[2]John Ring LaMontagne Center for Infectious Disease, The University of Texas at Austin, Austin, TX, United States.

[#] Present address: Human Biology Division, Fred Hutchinson Cancer Center, Seattle, WA, United States.

*** Correspondence:**
Shelley M. Payne
payne@utexas.edu

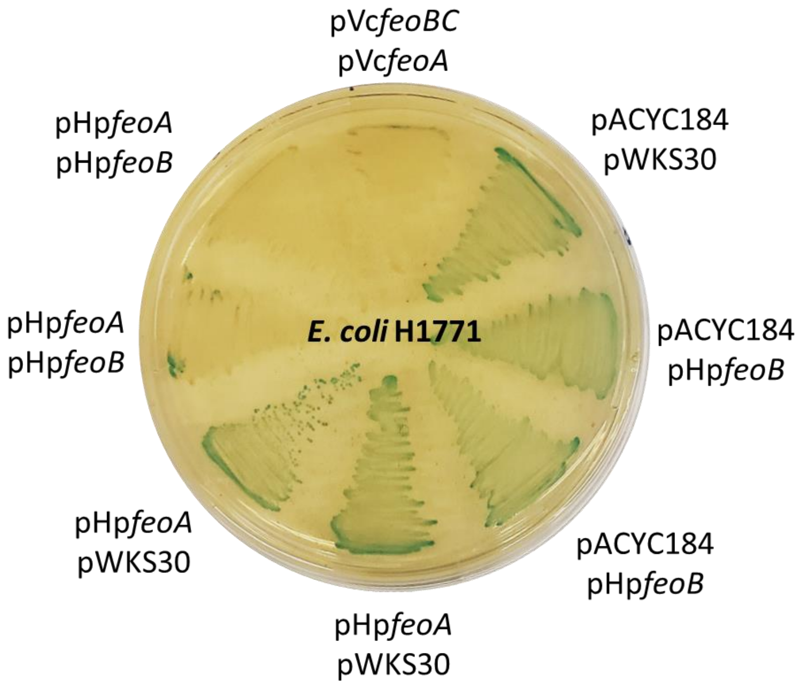

Supplementary Figure 1. Assessment of Feo function of HpFeoAB using *E. coli* H1117 as a proxy. When cultured in the presence of X-gal and 100 µM $FeSO_4$, *E. coli* H1771 colonies are white when transformed with a functional iron transport system and blue when there is no iron transport. In this case, *E. coli* H1771 was transformed with combinations of plasmids carrying the H. pylori feo genes in duplicates (pHpfeoA and pHpfeoB as indicated) or one *feo* gene together with the empty vector for the other gene (either pACYC184 for *HpfeoA* or pWKS30 for *HpfeoB*). The *V. cholerae feo* operon split in two vectors (pVcfeoA and pVcfeoBC) was used as a positive control, and the corresponding empty vectors for both constructs (pACYC184 and pWKS30) as a negative control. Only the combination of *HpfeoA* and *HpfeoB* led to a positive result in this assay, which is consistent with the results obtained with *V. cholera* EPV6 as shown in the main text.

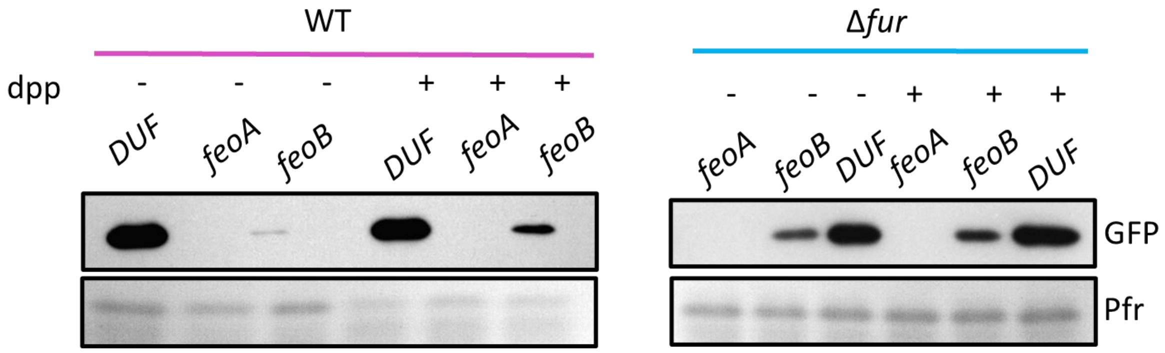

Supplementary Figure 2. Effect of dpp-induced iron starvation on GFP synthesis in the WT and Δ*fur H. pylori* backgrounds. Immunoblot analysis against GFP from the *gfp*-based transcriptional reporters in *H. pylori* WT and Δ*fur* after 48 h with (+) or without (-) 60 µM dpp addition. Ferritin (Pfr, shown in the bottom row from a Coomassie-stained gel) was used as a control for protein load.

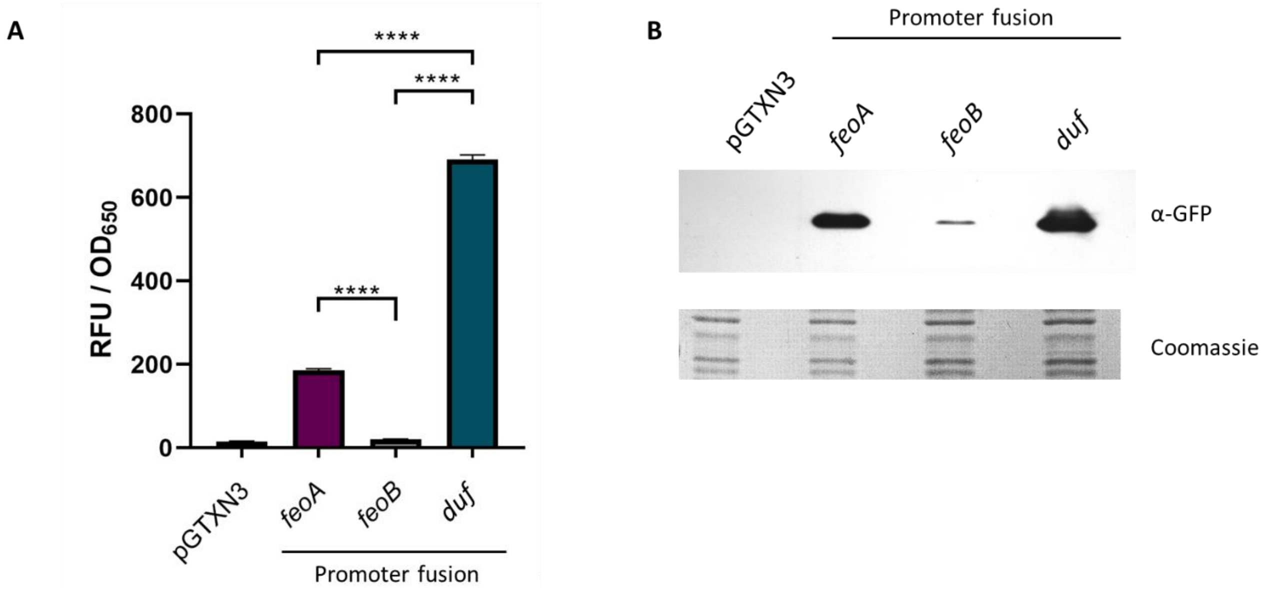

Supplementary Figure 3. The sequences upstream of *feoA*, *feoB*, and *duf* have promoter activity of different strength. (**A**) Fluorescence measurements of *E. coli* MFT-5 (*fur*-null strain) transformed with each of the promoter::*gfpmut3* fusions. pGTXN3 corresponds to the empty vector carrying the promoterless *gfpmut3* gene. Cells from mid-log phase cultures were harvested and washed with PBS to then measure the fluorescence in relative units (RFU) on a SpectraMax M3 (λ_{Ex} = 500 nm, λ_{Em} = 513 nm); these values were normalized by the absorbance at 650 nm (OD_{650}). The plotted values correspond to the means calculated from three independent biological replicates with three technical replicates (loaded wells) each. Error bars represent standard deviations. Statistical significance was inferred through two-tailed Student's t tests between means, and differences that were statistically significant are indicated (**** for P<0.0001). Statistical analyses and the bar graph were generated with GraphPad Prism v9.5.0. (**B**) Immunoblot analysis against GFP of the same samples used in panel A. The bottom row shows a control section of the same samples stained with Coomassie Blue R250 as a control for protein load.

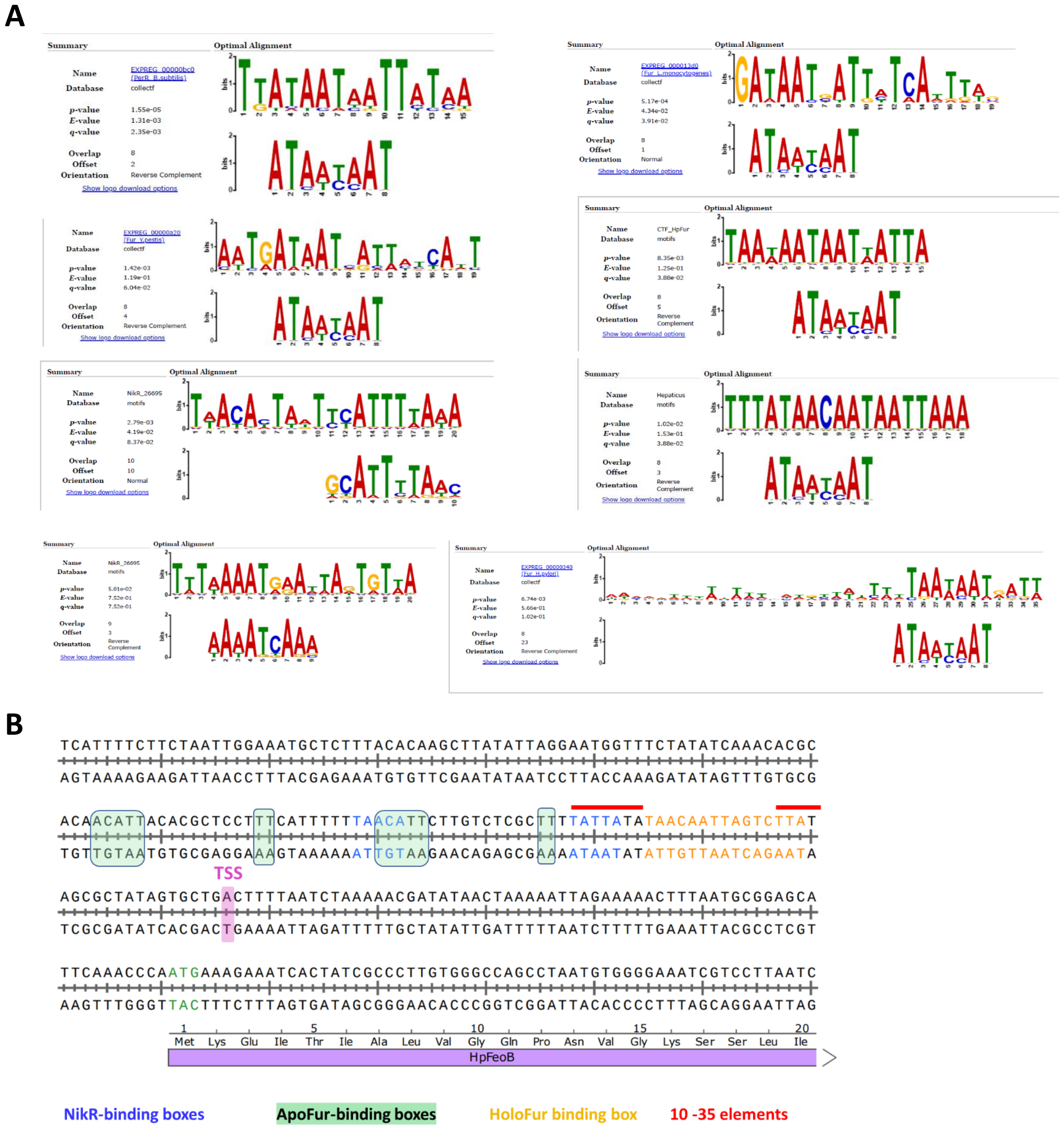

Supplementary Figure 4. Fur and NikR binding motifs in *feoB* promoters. (**A**) LOGO representations of significant matches found using the XSTREME algorithm in the *feoB* promoter alignment (query, bottom lane of each match) against experimentally validated motifs deposited in the CollecTF database and user-provider motifs (subject, lane of each match). (**B**) Proposed architecture of transcription regulatory elements in the *HpfeoB* promoter.

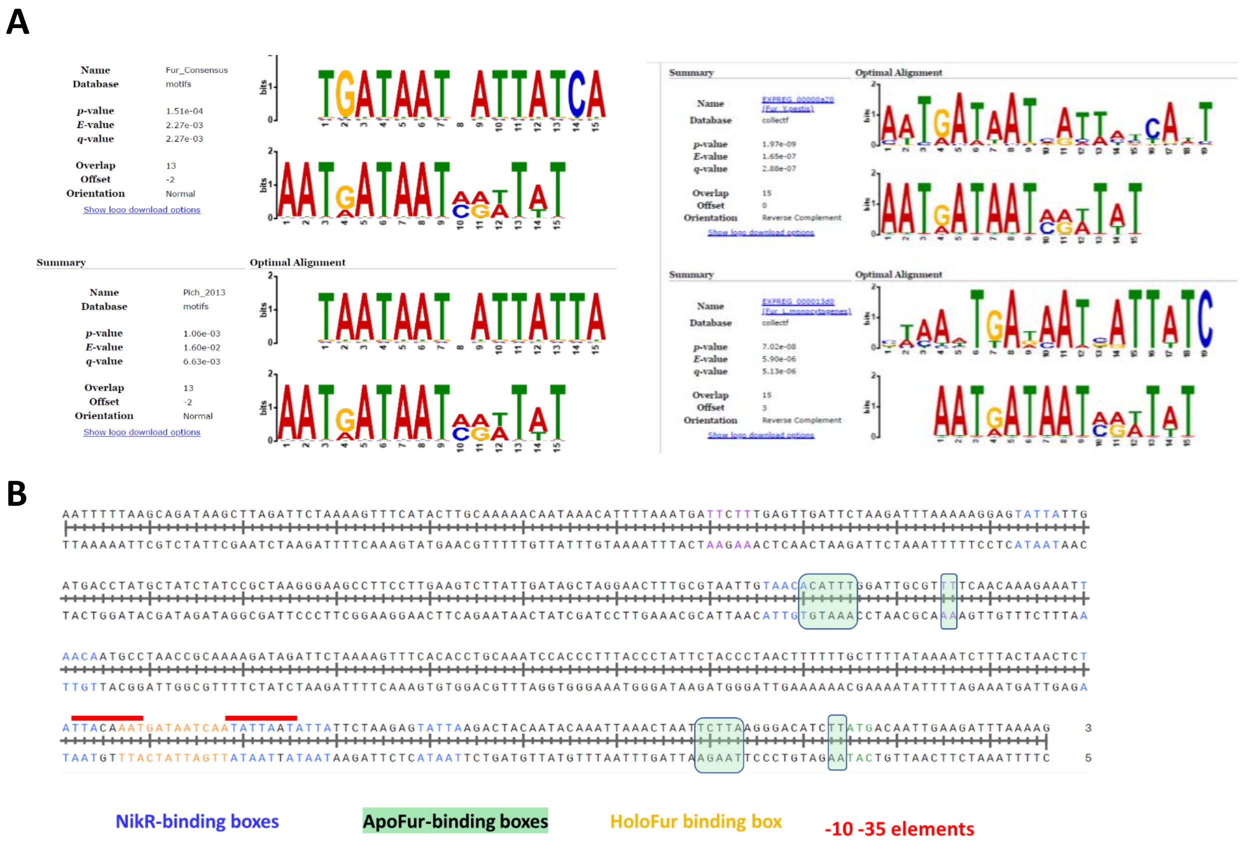

Supplementary Figure 5. Fur and NikR binding motifs in *feoAB* operon promoters. (**A**) LOGO representations of significant matches found using the XSTREME algorithm in the *feoAB* operon promoter alignment (query, bottom lane of each match) against experimentally validated motifs deposited in the CollecTF database and user-provider motifs (subject, lane of each match). (**B**) Example of the proposed architecture of transcription regulatory elements in the *feoAB* promoter of *H. canadensis*.

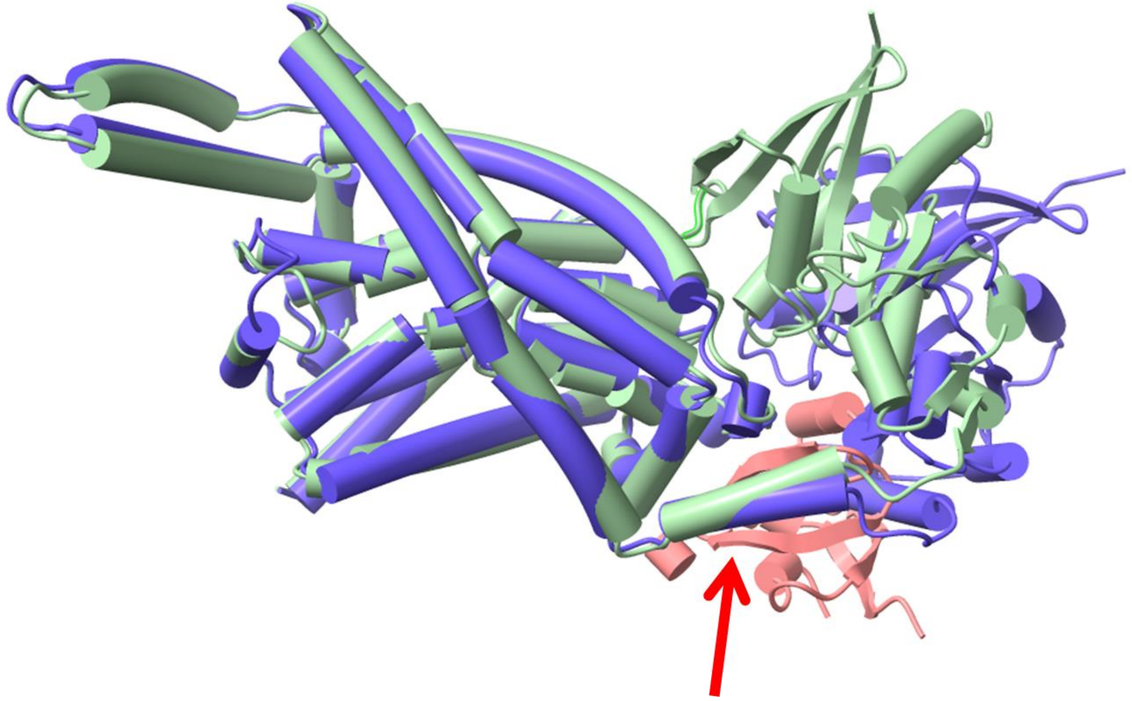

Supplementary Figure 6. AlphaFold model for HpFeoB shows a flexible structure around a hinge that locks upon HpFeoA binding. The cylinder representation of AlphaFold models for HpFeoB alone (blue) and HpFeoB bound to HpFeoA (green with HpFeoA in pink) are aligned to show the structural changes induced by HpFeoA binding. HpFeoB is predicted to have a flexible region in a hinge about the residue Ala208, indicated with a red arrow.

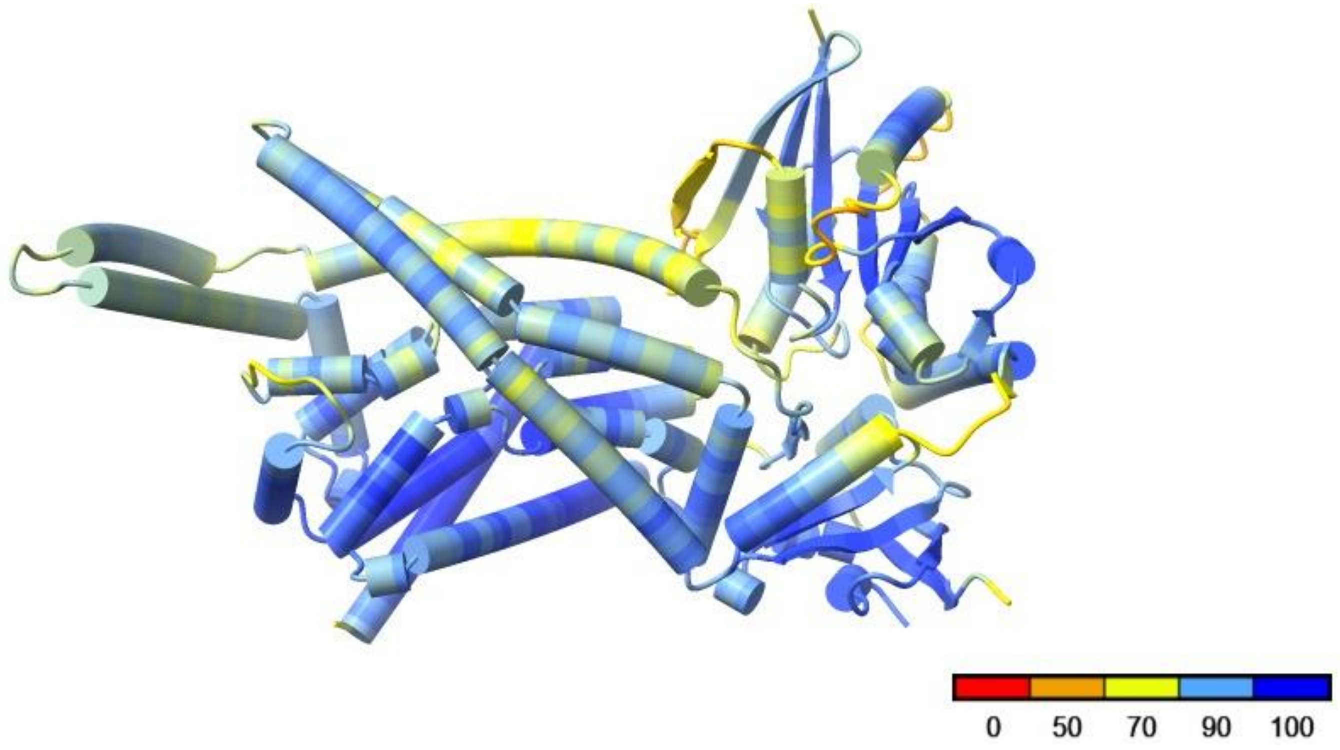

Supplementary Figure 7. 3D AlphaFold model of the HpFeoA-HpFeoB interaction colored according to the predicted local distance difference test (pLDDT) as a measure of per-residue estimate of confidence. The color code for the pLDDT score is shown in the bottom right corner. Regions with pLDDT between 70 – 90 are considered a generally good prediction. pLDDT scores greater than 90 are defined as high accurately.
